# Biochemical investigations using mass spectrometry to monitor JMJD6-catalysed hydroxylation of multi-lysine containing bromodomain-derived substrates†

**DOI:** 10.1039/d4cb00311j

**Published:** 2025-02-24

**Authors:** Thomas P. Corner, Eidarus Salah, Anthony Tumber, Lennart Brewitz, Christopher J. Schofield

**Affiliations:** a Chemistry Research Laboratory, Department of Chemistry and the Ineos Oxford Institute for Antimicrobial Research, University of Oxford 12 Mansfield Road Oxford OX1 3TA UK lennart.brewitz@chem.ox.ac.uk christopher.schofield@chem.ox.ac.uk

## Abstract

Jumonji-C domain-containing protein 6 (JMJD6) is a human 2-oxoglutarate (2OG)/Fe(ii)-dependent oxygenase catalysing post-translational C5 hydroxylation of multiple lysine residues, including in the bromodomain-containing proteins BRD2, BRD3 and BRD4. The role(s) of JMJD6-catalysed substrate hydroxylation are unclear. JMJD6 is important in development and JMJD6 catalysis may promote cancer. We report solid-phase extraction coupled to mass spectrometry assays monitoring JMJD6-catalysed hydroxylation of BRD2–4 derived oligopeptides containing multiple lysyl residues. The assays enabled determination of apparent steady-state kinetic parameters for 2OG, Fe(ii), l-ascorbate, O_2_ and BRD substrates. The JMJD6 *K*^app^_m_ for O_2_ was comparable to that reported for the structurally related 2OG oxygenase factor inhibiting hypoxia-inducible factor-α (FIH), suggesting potential for limitation of JMJD6 activity by O_2_ availability in cells, as proposed for FIH and some other 2OG oxygenases. The new assays will help development of small-molecule JMJD6 inhibitors for functional assignment studies and as potential cancer therapeutics.

## Abstract 2

High-throughput mass spectrometric assays monitoring JMJD6-catalysed hydroxylation of multi-lysine containing BRD2–4 derived oligopeptides are reported. Kinetic studies reveal the dependence of JMJD6 catalysis on O_2_ availability.

## Graphical abstract

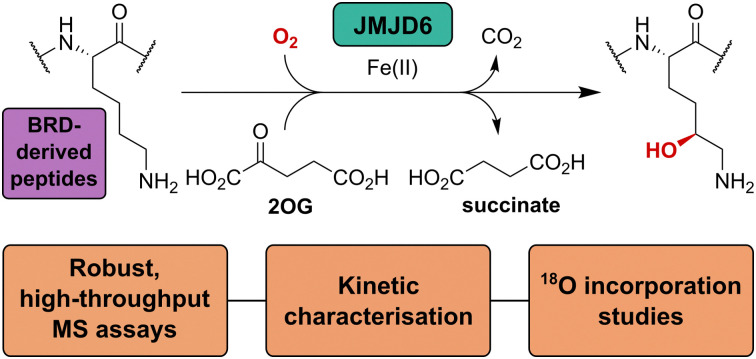

## Introduction

Non-heme Fe(ii)- and 2-oxoglutarate (2OG)-dependent oxygenases are ubiquitously distributed across almost all kingdoms of life.^1^ They typically utilise dioxygen (O_2_) and 2OG as co-substrates, and Fe(ii) as a cofactor, to catalyse two electron oxidations of their ‘prime’ substrates.^2,3^ The ∼70 human 2OG oxygenases identified have important biological roles, including in hypoxia sensing,^4,5^ collagen biosynthesis,^6^ epigenetic regulation,^7^ nucleic acid repair,^8^ and metabolism.^9,10^ The largest subfamily of human 2OG oxygenases are the Jumonji-C domain-containing 2OG oxygenases (JmjC oxygenases),^11^ the majority of which are histone *N*^*ε*^-methyl lysyl demethylases (KDMs)^12^ that catalyse *N*^*ε*^-methyl lysyl residue demethylation *via* methyl group C-hydroxylation, leading to release of formaldehyde as a co-product.^13^ Some JmjC demethylases also catalyse *N*-methyl arginyl demethylations and, potentially, other reactions.^14–17^

JmjC oxygenases have been identified that catalyse protein and nucleic acid hydroxylations,^11,18^*e.g.*, factor inhibiting hypoxia-inducible factor-α (FIH) which is reported to catalyse aspartate-,^19,20^ asparagine-,^4,5,19^ histidine-,^21^ leucine-^22^ and tryptophan-residue hydroxylations.^23,24^ Along with the non-JmjC 2OG-dependent hypoxia-inducible factor-α (HIF-α) prolyl hydroxylase domain-containing proteins 1–3 (PHD1–3), FIH is involved in regulating the cellular response to limiting O_2_ availability (*i.e.*, hypoxia). FIH catalyses hydroxylation of a specific asparagine residue within the C-terminal activation domain (CTAD) of HIF-α isoforms (Asn803 in human HIF-1α), a post-translational modification that hinders the interactions of α,β-HIF with histone acetyl transferases.^4,5^ Severe hypoxia inhibits FIH catalysis, resulting in enhanced HIF-α CTAD-promoted expression of HIF target genes.^4,19^ The kinetic and active site properties of FIH and the PHDs are proposed to reflect their roles as hypoxia sensors.^25–28^

**Fig. 1:**
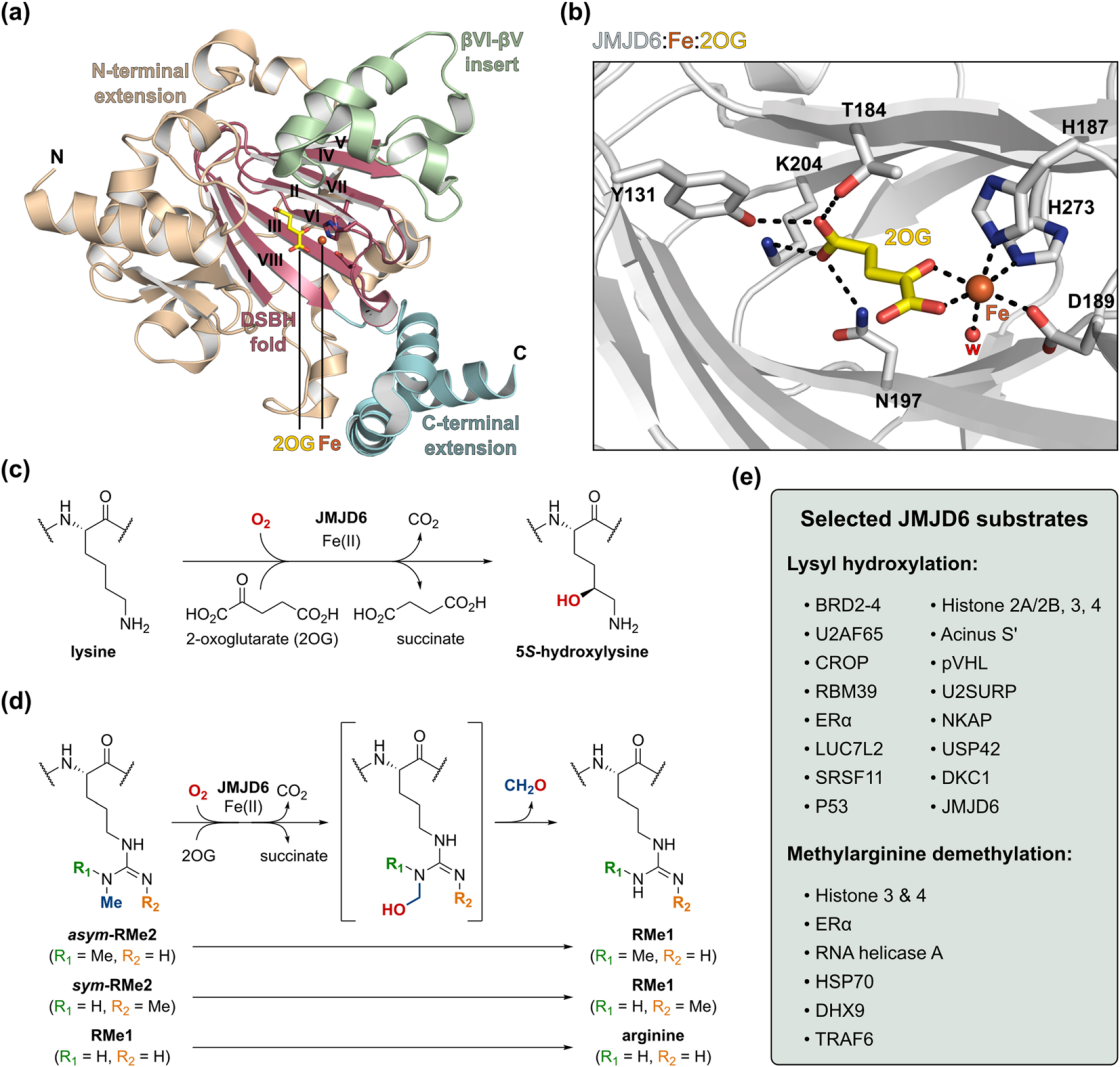
The human 2-oxoglutatarate (2OG)- and Fe(ii)-dependent oxygenase Jumonji-C domain-containing protein 6 (JMJD6). (a) View from a reported JMJD6_1–343_:Fe:2OG complex structure (PDB ID: 6DGY^36^), highlighting the secondary structure elements of JMJD6, *i.e.*, the double-stranded beta-helix (DSBH) core fold (magenta), the βVI-βV insert (green) and the C- and N-terminal extensions (blue and ochre, respectively). Roman numerals (I–VIII) correspond to the eight β-sheets of the DSBH fold. (b) Active site view of human JMJD6_1–343_ (light-grey) in complex with 2OG (yellow) and Fe (orange). w: water. (c) and (d) JMJD6 is reported to catalyse (c) lysyl C5 hydroxylation,^30,36^ and (d) the *N*-demethylation of mono- and symmetric/asymmetric di-methylated arginine residues.^34^ (e) Selected reported JMJD6 substrate proteins.^29,31–34,36,40–45^ See ESI,† for acronym definitions.

Like FIH, Jumonji-C domain-containing protein 6 (JMJD6) is a JmjC oxygenase that catalyses post-translational protein hydroxylations; however, unlike FIH, at the C5 position of protein lysine residues to give products with the (2*S*,5*S*) configuration (Fig. 1a–c).^29,30^ Biochemical and cell-based studies have suggested that JMJD6 may also catalyse the *N*-demethylation of mono- and di-methylated arginine residues (Fig. 1d and e),^31–33^*e.g.*, within histones H3 and H4.^34,35^ However, studies with isolated recombinant JMJD6 and *N*,*N*-dimethylarginine-bearing H3, H4 and HSP70 fragment peptides have been unable to support the proposed JMJD6 demethylase activity;^29,36–38^ it has also been reported that JMJD6 gene deletion does not affect histone H4 arginine-3 *N*-methylation levels in cells.^39^ Thus, the functional assignment of JMJD6 as a ‘direct’ *N*-methyl arginine demethylase requires further investigation.^38^

The available cellular evidence indicates that JMJD6 catalyses the hydroxylation of lysine residues in at least 50 proteins, including *e.g.*, histones,^45^ splicing regulatory (SR) proteins (*e.g.*, U2AF65,^29^ LUC7L2,^29^ RBM39^36^), the tumour suppressor protein p53,^36,41^ the von Hippel-Lindau protein (pVHL),^39^ and bromodomain-containing protein 2 (BRD2), BRD3 and BRD4 (Fig. 1e).^40^ Cellular studies have also revealed that many sites of JMJD6-catalysed lysyl hydroxylation are located within protein domains that contain sequences enriched with multiple positively charged lysine and arginine residues.^40^ For instance, the unstructured lysine-rich basic residue enriched interaction domains (BIDs) of BRD2–4 have been shown to be extensively hydroxylated by JMJD6; 19 sites of JMJD6-catalysed lysine hydroxylation are reported on BRD4, including on adjacent residues.^40^ The observed apparent protein substrate promiscuity of JMJD6 is precedented by the many validated substrates of the JmjC hydroxylase FIH, which also catalyses hydroxylation of proximate residues in some proteins,^28,46^ and those of the non-JmjC 2OG oxygenase aspartate/asparagine β-hydroxylase (AspH).^47–49^ The apparent promiscuity of JMJD6, FIH, and AspH contrasts with the apparently narrow substrate scope of the human JmjC 2OG oxygenases JMJD5^50,51^ and JMJD7,^52,53^ and the non-JmjC 2OG-dependent ribosomal oxygenases MYC-induced nuclear antigen (MINA53) and nucleolar protein 66 (NO66).^54^

The reported biological roles of JMJD6 are pleiotropic, possibly reflecting its broad substrate scope.^55^ The deletion of the *JMJD6* gene in mice is associated with malformations of vital organs, causing peri- and neo-natal lethality, indicating that JMJD6 may be critical in organ development during embryogenesis.^56–60^ JMJD6 catalysis is also reported to regulate pre-mRNA splicing of multiple protein targets, including potentially itself,^61^*via* SR protein hydroxylation.^29,36^ Lysine residue hydroxylation of the SR protein U2AF65 by JMJD6 is proposed to modulate the alternative splicing of *inter alia* vascular endothelial growth factor receptor 1 (FLT1)^39^ and ferrochelatase (FECH).^62^ Cellular and animal model studies have indicated that JMJD6 may have roles in epigenetic regulation,^34,45^ angiogenesis^42^ and haematopoiesis.^63^ Additionally, cellular studies have found that (aberrant) JMJD6 activity may promote cancer development and/or progression,^41,64–67^ for instance, JMJD6-catalysed p53 hydroxylation is proposed to enhance colon carcinogenesis,^41^ whilst JMJD6-catalysed hydroxylation of splicing regulatory proteins (*e.g.*, U2AF65) regulates expression of androgen receptor splice variant 7 (AR-V7), causing resistance to androgen receptor (AR) antagonists used for the treatment of prostate cancer.^67^ JMJD6 is thus a current medicinal target for prostate cancer treatment.^67^

The lysine-rich regions in several JMJD6 substrates are reported to be involved in intracellular condensate formation, including in BRD4.^68^ JMJD6 is also proposed to regulate transcriptional pause-release through its interactions with BRD4.^35^ The extent of JMJD6-catalysed BRD4 hydroxylation has been observed to vary in an O_2_ concentration-dependent manner in cells, suggesting the possibility that JMJD6 may be involved in control of intracellular partitioning processes and/or gene expression in response to hypoxia and/or additional physiological stresses.^40^ Notably, JMJD6 is up-regulated by hypoxia,^42^ in a manner similar to that observed for the hypoxia sensing prolyl hydroxylases PHD2 and PHD3.^69,70^

Recently, we reported matrix-assisted laser desorption/ionization mass spectrometry (MALDI-MS) end-point assays that enabled biochemical characterisation of the JMJD6-catalysed hydroxylation of multiple substrates, including histones H3 and H4, as well as the SR proteins U2AF65, LUC7L2 and RBM39.^36^ However, the application of these JMJD6 assays for kinetic analyses, important to analyse the 2OG and O_2_ dependence of JMJD6, and inhibition studies is limited by their requirement for relatively high enzyme (10 μM) and substrate concentrations (100 μM), and by their relatively low throughput.

By contrast with MALDI-MS, solid-phase extraction coupled to mass spectrometry (SPE-MS) enables direct, label-free quantification of enzyme catalysis, typically with high sensitivity and high signal-to-noise ratios; thus, relatively low concentrations of enzyme and substrate(s) are required.^71,72^ The SPE-MS assay setup is semi-automated, enabling on-line measurement of enzymatic reaction progress. SPE-MS assays have been successfully employed in kinetic and inhibition studies of enzymes, including JmjC oxygenases, by measuring the mass shift associated with the hydroxylation (*i.e.*, +16 Da) or demethylation (*i.e.*, −14 Da) of peptide, small-molecule and polynucleotide substrates.^49,71–78^

Here, we report the development of SPE-MS assays that monitor the JMJD6-catalysed C5 lysyl hydroxylation of peptide fragments derived from the sequences of BRD2, BRD3 and BRD4.^40^ Studies using isotopically labelled ^18^O_2_ gas revealed that oxygen incorporated into the hydroxylated BRD4 fragment peptide originates predominantly from atmospheric dioxygen. The assays were suitable for determining the kinetic parameters of JMJD6 for 2OG, Fe(ii), l-ascorbate, O_2_ and multi-lysine containing BRD2–4-derived oligopeptide substrates. Although further studies are required, the results indicate that, at least with the tested substrates, JMJD6 hydroxylation activity may be limited by O_2_ availability in a manner similar to FIH.

## Results and discussion

### Development of SPE-MS assays for monitoring JMJD6 catalysis

To develop robust assays for the biochemical characterisation of JMJD6 catalysis, we initially investigated whether reported protein fragments used to investigate JMJD6-catalysed hydroxylation of SR proteins in MALDI-based assays,^36^*e.g.*, LUC7L2_267–278_, U2AF65_30–46_, CROP_311–325_, SRSF11_286–298_ and RBM39_31–42_, are suitable substrates for SPE-MS assays. However, these peptides exhibited poor retention on SPE cartridges, likely due to their high basicity. We thus designed 40-mer peptide fragments with reduced basicity derived from homologous sequences (>75% sequence identity) within the lysine-rich BIDs of BRD2, BRD3 and BRD4, *i.e.*, BRD2_520–559_ (LAELQEQLRAVHEQLAALSQGPISKPKRKREKKEKKKKRK), BRD3_463–502_ (LAELQEQLKAVHEQLAALSQAPVNKPKKKKEKKEKEKKKK), and BRD4_511–550_ (LAELQEQLKAVHEQLAALSQPQQNKPKKKEKDKKEKKKEK).^40^ These peptides were prepared by solid-phase peptide synthesis (SPPS) and tested for retention on SPE cartridges. The results revealed that all three BRD-derived peptides were efficiently retained on a C4 SPE cartridge, enabling their quantification using SPE-MS.

**Fig. 2:**
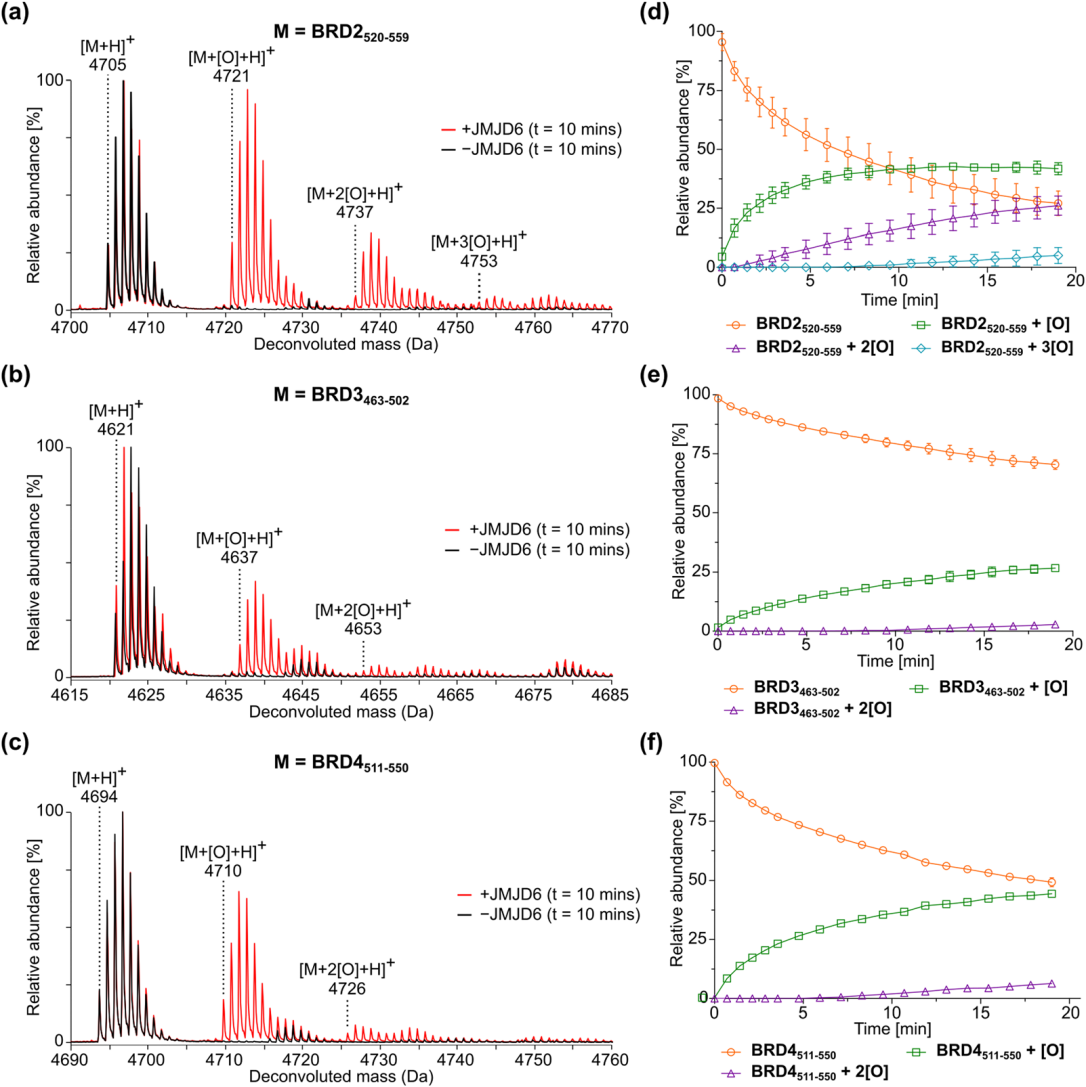
JMJD6-catalysed hydroxylation of homologous 40 mer peptides derived from bromodomain-containing proteins BRD2, BRD3, and BRD4. (a)–(c) Red MS spectra: JMJD6-catalysed hydroxylation after 10 min of (a) BRD2_520–559_, (b) BRD3_463–502_, and (c) BRD4_511–550_. Black MS spectra: no enzyme controls. (d)–(f) JMJD6-catalysed hydroxylation of (d) BRD2_520–559_, (e) BRD3_463–502_, and (f) BRD4_511–550_ showing the relative abundance of the non-hydroxylated substrate (orange circles), mono-hydroxylated product (green squares), di-hydroxylated product (purple triangles) and tri-hydroxylated product (light blue diamonds) over time. Data are means of three independent runs (*n* = 3; means ± SD). Hydroxylation reactions were performed as described in the Experimental Section using full-length His_6_-JMJD6 (0.05 μM), 2OG (200 μM), (NH_4_)_2_Fe(SO_4_)_2_·6H_2_O (FAS; 2 μM), l-ascorbic acid (LAA; 100 μM) and BRD2_520–559_, BRD3_463–502_ or BRD4_511–550_ substrate (2 μM) in Tris buffer (50 mM, pH 7.5).

Time-course studies employing 0.05 μM isolated recombinant JMJD6 (Fig. S1, ESI†) and 2 μM BRD2_520–559_, BRD3_463–502_ or BRD4_511–550_ (*i.e.*, an enzyme/substrate ratio of: 1/40) confirmed that the three BRD-derived peptides are efficiently hydroxylated by isolated recombinant JMJD6 and that the +16 Da mass shift(s) associated with their hydroxylation(s) can be directly monitored using SPE-MS (Fig. 2a–f). Importantly, no evidence for substrate oxidation (*i.e.*, +16 Da mass shifts) was accrued under the SPE-MS assay conditions in the absence of JMJD6. JMJD6 likely catalyses sequential hydroxylations of the BRD2_520–559_, BRD3_463–502_ and BRD4_511–550_ substrates, as evidenced by the observation of up to three +16 Da mass shifts. During the initial time-course reactions, three hydroxylation products were observed for BRD2_520–559_, and two hydroxylation products were observed for both BRD3_463–502_ and BRD4_511–550_ (Fig. 2). These observations support reported work showing that the BIDs of the BRD2–4 proteins are poly-hydroxylated by JMJD6 in cells.^40^

The sum of the ion counts of the non-hydroxylated substrate peptides and the hydroxylated product peptides was approximately constant over time for all the three BRD-derived JMJD6 substrates investigated in this study (Fig. S2, ESI†). This observation implies that both substrates and products are retained on the SPE cartridge with approximately equal efficiencies under the employed conditions and that their ionisation properties are similar. Thus, JMJD6 catalysis can be quantified by direct comparison of the ion counts of substrate and product peptides, without the need for an internal standard in the assay mixture. Notably, the required JMJD6 and substrate concentrations used were 200- and 50-fold less than those used in the reported MALDI-TOF JMJD6 kinetic studies,^36^ reflecting the high sensitivity of the SPE-MS JMJD6 assays.

### Steady-state kinetic parameters for JMJD6 and BRD derived substrates

**Fig. 3:**
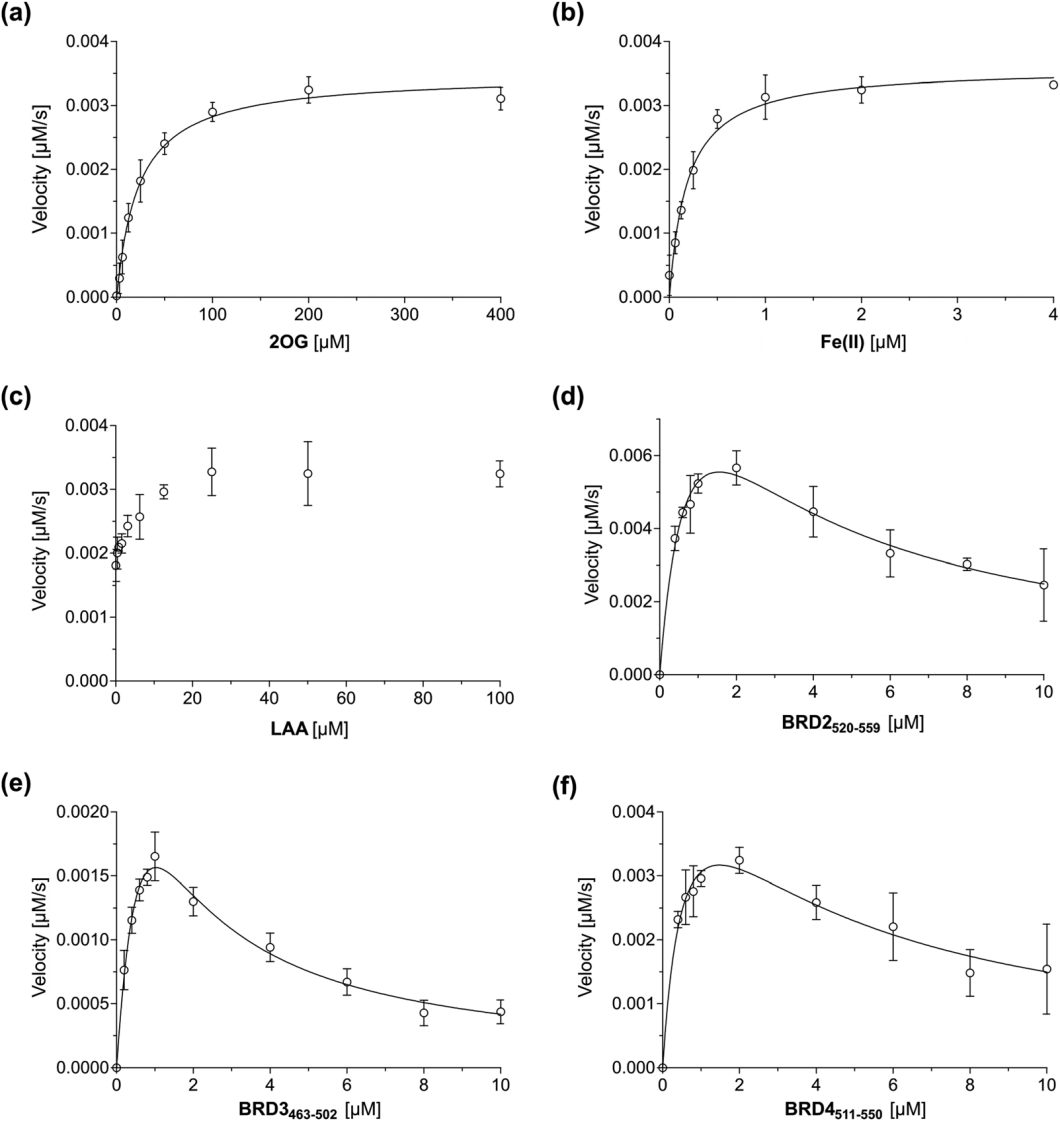
Michaelis–Menten plots used to determine steady-state kinetic parameters for JMJD6. Kinetic parameters of JMJD6 determined for: (a) 2OG; (b) Fe(ii); (c) LAA; (d) BRD2_520–559_; (e) BRD3_463–502_; (f) BRD4_511–550_. Data are means of three independent runs (*n* = 3; means ± SD). The results are summarized in Table 1. SPE-MS assays were performed as described in the Experimental Section using full-length His_6_-JMJD6 (0.05 μM). BRD4_511–550_ was used as the JMJD6 substrate to determine the apparent kinetic parameters for 2OG, Fe(ii) and LAA. Time-course data and initial velocities are shown in the ESI.†

Having established that the JMJD6-catalysed hydroxylation of BRD2_520–559_, BRD3_463–502_ and BRD4_511–550_ can be efficiently monitored using SPE-MS, kinetic studies on isolated recombinant full-length His_6_-JMJD6 (Fig. S1, ESI†) were initiated. SPE-MS assays monitoring the JMJD6-catalysed hydroxylation of BRD4_511–550_ in the presence of likely saturating (co)substrate concentrations (*i.e.*, >2 × *K*^app^_m_) enabled the determination of both the apparent maximum velocities (*v*^app^_max_) and the apparent Michaelis constants (*K*^app^_m_) of JMJD6 for 2OG, Fe(ii), LAA and BRD4_511–550_ (Fig. 3a–d). Note that kinetic analyses of JMJD6 are complicated by its oligomeric nature.^37,79^

BRD4_511–550_ was used as the preferred substrate for the kinetic studies, because the initial time-courses indicated BRD4_511–550_ is mono-hydroxylated more efficiently than BRD3_463–502_ whilst showing substantially lower levels of di-hydroxylation and no tri-hydroxylation by comparison with BRD2_520–559_ (Fig. 2), the presence of which may complicate data analysis. Indeed, negligible levels of BRD4_511–550_ hydroxylations beyond mono-hydroxylation were observed during the initial time-period (*i.e.*, 90 s) of all SPE-MS assays analysed for determining the JMJD6 kinetic parameters.

Turnover numbers (*k*^app^_cat_) and specificity constants (*k*_cat_/*K*_m_) were calculated from *v*^app^_max_ and *K*^app^_m_ values assuming that the concentration of active JMJD6 equals the total enzyme concentration used in the assay (*i.e.*, 0.05 μM). Note, that this assumption may not be fully valid, including because JMJD6 can homo-oligomerize^80,81^ and because JMJD6 is reported to catalyse self-hydroxylation, which may affect its catalytic activity.^43^ However, a sufficiently tight-binding or irreversible JMJD6 inhibitor suitable for active-site titrations has not yet been reported.

**Table 1:** Steady-state kinetic parameters of JMJD6^a^

	(Co-)substrate/cofactor	*v* ^app^_max_ [nM s^−1^]	*k* ^app^_cat_ b [s^−1^]	*K* ^app^_m_ [μM]	*k*_cat_/*K*_m_ [mM^−1^ s^−1^]
i	2OG^c^^d^	3.5 ± 0.1	0.070 ± 0.002	23.3 ± 2.5	3.0 ± 0.4
ii	Fe(ii)^c^^d^	3.6 ± 0.1	0.072 ± 0.002	0.19 ± 0.02	380 ± 50
iii	BRD2_520–559_^d^	11.9 ± 2.5	0.24 ± 0.05	0.89 ± 0.33	270 ± 110
iv	BRD3_463–502_^d^	5.2 ± 1.3	0.10 ± 0.02	1.2 ± 0.4	87 ± 38
v	BRD4_511–550_^d^	5.8 ± 1.2	0.12 ± 0.02	0.62 ± 0.26	190 ± 80
vi	O_2_^c^^e^	6.2 ± 0.3	0.12 ± 0.01	69.8 ± 11.4	1.7 ± 0.4

a Determined using 0.05 μM full-length His_6_-JMJD6 in buffer (50 mM Tris, pH 7.5), as described in the Experimental section. Data are means of three independent runs (*n* = 3; mean ± SD).

b *k* ^app^_cat_ values were calculated from *v*^app^_max_ values assuming that the concentration of active JMJD6 equals the total enzyme concentration (*i.e.*, 0.05 μM).

c Kinetic parameters were determined using BRD4_511–550_ (2 μM) as the substrate.

d SPE-MS assays were performed at ambient temperature and run for ∼20 min.

e SPE-MS assays were performed at 37 °C and quenched after 60 or 90 s.

The *K*^app^_m_ value of JMJD6 for 2OG was ∼23 μM, which is in the range of that obtained with the MALDI-TOF assay using LUC7L2_267–278_ as the substrate (∼31 μM).^36^ This value is relatively high compared to 2OG *K*^app^_m_ values reported for most other human 2OG oxygenases that have been determined using SPE-MS assays (Table 2), *e.g.*, AspH (∼0.2 μM)^49^ and the JmjC domain-containing oxygenases JMJD5 (∼0.3 μM),^74^ FIH (∼0.8 μM),^82^ KDM4C (∼0.08 μM),^72^ MINA53 (∼3 μM),^77^ and NO66 (∼0.8 μM).^77^ However, the *K*^app^_m_ value of JMJD6 for 2OG is ∼6-fold less than that reported for isolated human γ-butyrobetaine hydroxylase (BBOX; ∼150 μM), although this value was determined using ^1^H NMR turnover assays,^83,84^ and so may not be directly comparable with that determined for JMJD6. Moreover, BBOX is a dimer and is reported to manifest cooperativity in substrate/co-substrate binding which may affect its *K*^app^_m_ value for 2OG,^85^ as may also be the case for JMJD6 given that it is oligomeric.^37,79^ The *K*^app^_m_ value of JMJD6 for 2OG is lower than reported cellular 2OG concentrations (0.5–1 mM),^86,87^ an observation which indicates that JMJD6 activity may not be limited by 2OG availability in cells; however, it is possible that the *K*^app^_m_ value of JMJD6 for 2OG varies with sub-cellular location and/or the substrate employed, and hence, that 2OG availability may limit the JMJD6-catalysed hydroxylation of substrates other than BRDs, given the relatively broad substrate scope of JMJD6.^36,40^

**Table 2:** Steady-state kinetic parameters of selected human 2OG oxygenases

Enzyme	2OG			Fe(ii)			Substrate		
	*k* ^app^_cat_ [s^−1^]	*K* ^app^_m_ [μM]	*k*_cat_/*K*_m_ [mM^−1^ s^−1^]	*k* ^app^_cat_ [s^−1^]	*K* ^app^_m_ [μM]	*k*_cat_/*K*_m_ [mM^−1^ s^−1^]	*k*_cat_ [s^−1^]	*K*_m_ [μM]	*k*_cat_/*K*_m_ [mM^−1^ s^−1^]
JMJD6^a^^b^	0.070 ± 0.002	23.3 ± 2.5	3.0 ± 0.4	0.072 ± 0.002	0.19 ± 0.02	380 ± 50	0.12 ± 0.02	0.62 ± 0.26	190 ± 80
JMJD5^a^^c^ ^74^	5.6 ± 0.2 × 10^−3^	0.29 ± 0.04	19.3 ± 5.8	5.0 ± 0.2 × 10^−3^	0.13 ± 0.02	38.5 ± 6.2	10 ± 2.7 × 10^−3^	0.87 ± 0.46	11.5 ± 6.3
AspH^a^^d^ ^49^	0.19 ± 0.03	0.60 ± 0.09	320 ± 70	0.19 ± 0.03	1.42 ± 0.16	130 ± 30	0.20 ± 0.03	1.19 ± 0.26	170 ± 50
FIH^a^^e^ ^82^	0.04 ± 0.01	0.8 ± 0.1	47.6 ± 12.5	n. r.	n. r.	n. r.	n. r.	n. r.	n. r.
PHD2^f^ ^88^	n. r.	0.35 ± 0.03	n. r.	n. r.	0.89 ± 0.07	n. r.	n. r.	7.3 ± 1.3	n. r.
KDM4C^a^^g^ ^72^	0.075 ± 0.001	2.6 ± 0.1	28.5 ± 1.3	n. r.	n. r.	n. r.	0.089 ± 0.004	5.8 ± 0.7	15.4 ± 1.9
BBOX^h^ ^83,84^	1.6 ± 0.1	153 ± 44	10 ± 3	n. r.	n. r.	n. r.	0.83	4.2	n. r.
MINA53^a^^i^ ^77^	n. r.	3.2 ± 0.6	n. r.	n. r.	0.5 ± 0.2	n. r.	n. r.	10.5 ± 5.5	n. r.
NO66^a^^j^ ^77^	n. r.	0.83 ± 0.09	n. r.	n. r.	0.014 ± 0.001	n. r.	n. r.	19.1 ± 6.3	n. r.

a Kinetics parameters were determined using SPE-MS.

b Using full-length His_6_-JMJD6 (0.05 μM) and BRD4_511–550_ as the substrate.

c Using JMJD5 (0.15 μM) and RSP6_128–148_ as the substrate.^74^

d Using AspH_315–758_ (0.1 μM) and a synthetic cyclic peptide based on human Factor X (hFX-CP_101–119_)^47^ as the substrate.^49^

e Using FIH (0.15 μM) and a HIF-1α C-terminal transactivation domain fragment (HIF-1α_789–822_)^25^ as the substrate.^82^

f Kinetic parameters were determined using a fluorescence resonance energy transfer assay using PHD2 (1.0 nM) and HIF-1α-derived biotin-DLEMLAPYIPMDDDFQL as the substrate.^88^

g Using KDM4C (0.5 μM) and ARTAQTARK(me3)STGGIA (a histone 3 K9(me3) derivative) as the substrate.^72^

h Kinetic parameters were determined using a ^1^H NMR turnover over assay using BBOX (0.05 μM) and γ-butyrobetaine (GBB) as the substrate.^83,84^

i Using MINA53_26–464_ (0.15 μM) and RPL27A_31–49_ as the substrate.^77^

j Using NO66_183–641_ (0.3 μM) and RPL8_205–224_ as the substrate.^77^ n. r., not reported. Note that in some cases, interpretation of kinetic parameters is complicated by the oligomeric nature of the 2OG oxygenases, including for JMJD6,^79^ BBOX,^85^ and MINA53/NO66.^89^

The *K*^app^_m_ of JMJD6 for Fe(ii) was ∼0.19 μM, which is in the range of those values reported for other 2OG oxygenases determined using SPE-MS assays (Table 2),^49,74,77^ and is likely indicative of a relatively high affinity of JMJD6 for Fe(ii), under catalytic conditions, at least compared to other 2OG oxygenases.

The JMJD6 assays were performed in the presence of LAA, the addition of which had a beneficial effect on JMJD6 catalysis. Although LAA was not essential for productive JMJD6 catalysis, the relative abundance of the mono-hydroxylated BRD4_511–550_ peptide appeared to plateau after ∼10 min at ∼15% in the absence of LAA (Fig. S5, ESI†). The ability of LAA to enhance the activity of multiple purified 2OG oxygenases, for reasons incompletely defined, is reported,^90^*e.g.*, for the procollagen prolyl hydroxylases^91^ and PHD2,^92–94^ to which LAA has been proposed to bind directly.^95^ In addition, LAA has been shown to improve SPE-MS assay robustness for 2OG oxygenases, including AspH and JMJD5; however, the presence of LAA was not found to be essential for efficient catalysis in these cases.^49,74^

Steady-state kinetic parameters for BRD2_520–559_ and BRD3_463–502_ were measured, then compared with those for BRD4_511–550_ (Fig. 3e and f). As with BRD4_511–550_, initial time periods were chosen to minimize levels of substrate di-hydroxylation (*i.e.*, 45 s for BRD2_520–559_ and 90 s for BRD3_462–502_), to facilitate data analysis. The SPE-MS assay results indicate that at high substrate concentrations (>2 μM), BRD2_520–559_, BRD3_463–502_ and BRD4_511–550_ inhibit JMJD6 catalysis (Fig. 3); such substrate inhibition is precedent with other 2OG oxygenases.^49,53,74^ Therefore, to calculate kinetic parameters of JMJD6 for the three peptide substrates, data were fitted using non-linear regression to an equation which accounts for substrate inhibition (*Y* = *v*^app^_max_·*X*/(*K*^app^_m_ + *X*·(1 + *X*/*K*_*i*_))). The results give a *k*_cat_/*K*_m_ value (∼190 mM^−1^ s^−1^) of BRD4_511–550_ which is ∼200–1000-fold greater than values reported for the SR protein substrate peptides LUC7L2_267–278_ (∼0.2 mM^−1^ s^−1^; Table 3), U2AF65_30–46_ (∼0.1 mM^−1^ s^−1^) and RBM39_31–42_ (∼0.8 mM^−1^ s^−1^), for which substrate inhibition was not reported.^36^ This difference indicates that BRD4_511–550_ may be a more efficient JMJD6 substrate than the reported SR-derived oligopeptides^36^ at a specific concentration, potentially due to the increased length of the BRD4_511–550_ oligopeptide used. The catalytic efficiency observed for the hydroxylation of BRD2_520–559_ (*k*_cat_/*K*_m_ ∼ 270 mM^−1^ s^−1^) was similar to that observed for BRD4_511–550_, whilst that observed for BRD3_463–502_ (*i.e.*, ∼90 mM^−1^ s^−1^) was ∼2-fold less than that for BRD4_511–550_. The combined results may, in part, reflect the observation that the hydroxylation levels of JMJD6 substrates vary in cells,^40^ as reported for AspH which, like JMJD6, also catalyses hydroxylation of a relatively large set of substrates (>100).^48^ It should be noted, however, that the hydroxylation kinetics of folded full-length protein substrates will likely differ from those of the tested peptide fragments, as precedented with other 2OG oxygenases, including FIH.^46^

**Table 3:** Steady-state kinetic parameters of JMJD6 for selected peptide substrates

	Peptide	Amino acid sequence	*k*_cat_ [s^−1^]	*K*_m_ [μM]	*k*_cat_/*K*_m_ [mM^−1^ s^−1^]
i	BRD2_520–559_^a^	LAELQEQLRAVHEQLAALSQGPISKPKRKREKKEKKKKRK	0.24 ± 0.05	0.89 ± 0.33	270 ± 110
ii	BRD3_463–502_^a^	LAELQEQLKAVHEQLAALSQAPVNKPKKKKEKKEKEKKKK	0.10 ± 0.02	1.2 ± 0.4	87 ± 38
iii	BRD4_511–550_^a^	LAELQEQLKAVHEQLAALSQPQQNKPKKKEKDKKEKKKEK	0.12 ± 0.02	0.62 ± 0.26	190 ± 80
iv	LUC7L2_267–278_^b^ ^36^	NPKRSRSREHRR	0.011	51.0 ± 4.6	0.22
v	U2AF65_30–46_^b^ ^36^	SRSRSRDRKRRSRSRDR	0.009	78.0 ± 15.0	0.12
vi	CROP_311–325_^b^ ^36^	SRDHKRSRSRERRRS	0.019	71.0 ± 7.2	0.27
vii	SRSF11_286–298_^b^ ^36^	RSKSPRRRRSHSR	0.008	41.0 ± 6.6	0.20
viii	RBM39_31–42_^b^ ^36^	RSKKRKKSKSRS	0.028	56.0 ± 9.6	0.40

a Kinetic parameters were determined using SPE-MS using full-length His_6_-JMJD6 (0.05 μM) and 2OG (200 μM).

b Kinetic parameters were determined using MALDI-TOF MS using His_6_-JMJD6_1–362_ (10 μM) and 2OG (500 μM). BRD: bromodomain-containing protein; LUC7L2: Luc7-like 2; U2AF65: U2 small nuclear RNA auxiliary factor 2; CROP: cisplatin resistance-associated protein; SRSF11: serine/arginine-rich splicing factor 11; RBM39: RNA-binding protein 39.

**Fig. 4:**
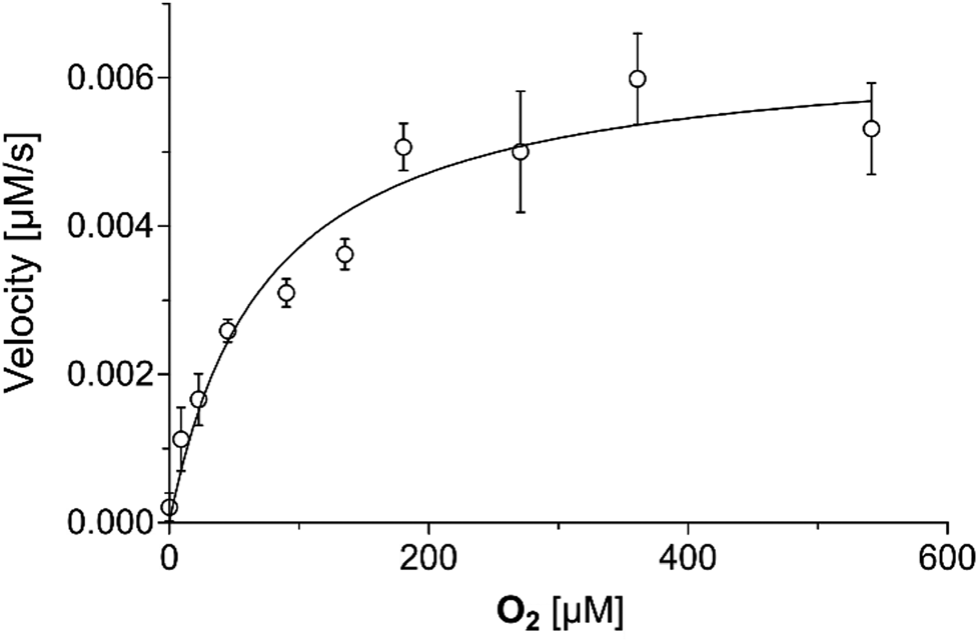
Michaelis–Menten plot used to determine the apparent steady-state kinetic parameters of JMJD6 for O_2_. The JMJD6/BRD4_511–550_*k*^app^_cat_ and *K*^app^_m_ for 2OG are 0.12 ± 0.01 s^−1^ and 74 ± 14 μM, respectively, as determined by non-linear regression. Data are means of three independent runs (*n* = 3; means ± SD). The results are summarized in Table 4. Assays were performed as described in the Experimental Section using full-length His_6_-JMJD6 (0.05 μM) and BRD4_511–550_ as the substrate (2 μM). Initial velocities are shown in the ESI.†

The dependency of the JMJD6-catalysed BRD4_511–550_ hydroxylation reaction on O_2_ availability was investigated next, including to inform on the potential of JMJD6 to serve as a hypoxia sensor (Fig. 4). Initial reaction velocities were calculated by performing the hydroxylation reactions under different partial pressures of O_2_, followed by quenching by the addition of formic acid (10%_v/v_) after 60 or 90 s; these time periods were carefully chosen to reduce levels of JMJD6-catalysed BRD4_511–550_ di-hydroxylation. The results reveal that the *K*^app^_m_ of JMJD6/BRD4_511–550_ for O_2_ was ∼74 μM and that JMJD6-catalysed BRD4_511–550_ hydroxylation was inhibited at low oxygen concentrations ([O_2_] < 2.5%), an observation which is consistent with reported cellular studies showing that the extent of BRD4 lysine hydroxylation is reduced in hypoxia.^40^

The *K*^app^_m_ value of JMJD6/BRD4_511–550_ for O_2_ is ∼2- and ∼4-fold greater those reported for type I human collagen prolyl 4-hydroxylase (CP4H-I; ∼40 μM)^26^ and KDM6B (∼20 μM),^96^ respectively, which, however, were not determined by SPE-MS, but by monitoring the release of radiolabelled [^14^C]CO_2_ from [1-^14^C]-2OG, which may compromise the direct comparability of these values (Table 4). The *K*^app^_m_ value of JMJD6 for O_2_ is in the range of those reported for phytanoyl-CoA dioxygenase (PHYH; ∼90 μM),^97^ which was determined using an oxygen consumption assay, and FIH (∼110 μM).^28^ The similar *K*^app^_m_ (O_2_) values for JMJD6 and FIH implies that *in vivo* O_2_ availability may influence JMJD6 activity in a similar manner to FIH.^25,28,98^ Note that the O_2_ dependency of JMJD6 may vary with its substrate, the substrate structure/fold, and/or the assay conditions. It is possible that the use of folded full-length substrate proteins could affect JMJD6 oligomerisation, resulting in conformational changes that modulate O_2_ availability or reaction at the active site.^37,79^ It is currently unclear if and to what extent limited O_2_ availability may alter the order of JMJD6-catalysed hydroxylations, including of BRDs.

**Table 4:** Steady-state kinetic parameters of selected 2OG oxygenases for O_2_

	Enzyme	*K*_m_ for O_2_ [μM]	*k*_cat_ [s^−1^]		Enzyme	*K*_m_ for O_2_ [μM]	*k*_cat_ [s^−1^]
i	JMJD6^a^	74 ± 14	0.12 ± 0.01	vii	KDM4C^g^ ^99^	158 ± 13	0.043 ± 0.001
ii	AspH^b^ ^49^	426 ± 73	0.23 ± 0.04	viii	KDM4E^h^ ^99^	197 ± 16	0.067 ± 0.001
iii	FIH^c^ ^28^	110 ± 30	0.56 ± 0.04	ix	KDM6A^i^ ^96^	180 ± 40	n. r.
iv	PHD2^d^ ^28^	460 ± 30	0.06 ± 0.01	x	KDM6B^j^ ^96^	20 ± 2	n. r.
v	PHD2^e^ ^28^	>450	0.028 ± 0.001	xi	CP4H-I^k^ ^26^	40	n. r.
vi	KDM4A^f^ ^100^	173 ± 23	n. r.	xii	PHYH^l^ ^97^	93 ± 43	0.016

a Using full-length His_6_-JMJD6 (0.05 μM) and BRD4_511–550_ as the substrate.

b Using AspH (0.1 μM) and hFX-CP_101–119_^47^ as the substrate.^49^

c Using FIH (5 μM) and HIF-1α_789–822_ as the substrate.^28^

d Using PHD2 (4 μM) and HIF-1α_556–574_ as the substrate.^28^

e Using PHD2 (4 μM) and HIF-1α_395–413_ as the substrate.^28^

f Using KDM4A (1 μM) and H3_1–15_K9me3 as the substrate.^100^

g Using KDM4C (3 μM) and H3_1–15_K9me3 as the substrate.^99^

h Using KDM4E (2.1 μM) and H3_1–15_K9me3 as the substrate.^99^

i Using KDM6A (1.5 μM) and H3_21–44_K27me3-Gly-Biotin as the substrate.^96^

j Using KDM6B (1.5 μM) and H3_21–44_K27me3-Gly-Biotin as the substrate.^96^

k Using CP4H-I and (Pro-Pro-Gly)_10_ as a substrate.^26^

l Using PHYH (50 μM) and isovaleryl-CoA as the substrate.^97^ n. r., not reported.

Under assay conditions similar to those employed for JMJD6, the *K*^app^_m_(O_2_) value reported for KDM4A (∼170 μM) is ∼2-fold greater that of JMJD6,^100^ whereas the *K*^app^_m_(O_2_) values reported for PHD2 (>450 μM)^28^ and AspH (∼430 μM)^49^ are ∼6-fold greater than that of JMJD6 (Table 4). The *K*^app^_m_(O_2_) value of JMJD6 is also less than those values reported for KDM4C (∼160 μM),^99^ KDM4E (∼200 μM)^99^ and KDM6A (∼180 μM),^96^ which were determined using oxygen consumption assays (for KDM4C and KDM4E) and by assaying the release of radiolabelled [^14^C]CO_2_ from [1-^14^C]-2OG (for KDM6A). The observation that the *K*^app^_m_(O_2_) value of JMJD6 is lower than that reported for PHD2,^28^ but similar to that for FIH,^28^ is of interest as the role of PHD2 in regulating gene expression in response to hypoxia is more defined than that of FIH,^5,101^ with PHD catalysis being more sensitive to O_2_ availability than FIH catalysis, as observed both in studies with isolated enzymes and in cells.^28,101,102^

### Origin of oxygen incorporated in the hydroxylated BRD4_511–550_ product

Although variations occur, a generalised mechanism has been proposed by which 2OG oxygenases typically catalyse substrate oxidation mediated by the active site bound Fe(ii) (Fig. S10, ESI†).^2,3^ Mechanistic studies have revealed that for hydroxylation reactions catalysed by FIH,^19^ PHD1^103^ and the procollagen prolyl hydroxylases (CPHs),^104^ the oxygen atom incorporated into hydroxylated products originates from atmospheric O_2_, with the other oxygen atom from O_2_ being incorporated into succinate.^105^ By contrast, bacterial and fungal dioxygenases have been identified that can incorporate substantial levels of water-derived oxygen, as well as O_2_-derived oxygen, into their hydroxylated products.^106–111^ In these examples, the exchange of oxygen from Fe-bound O_2_ for that from water is proposed to occur during the catalytic cycle.

**Fig. 5:**
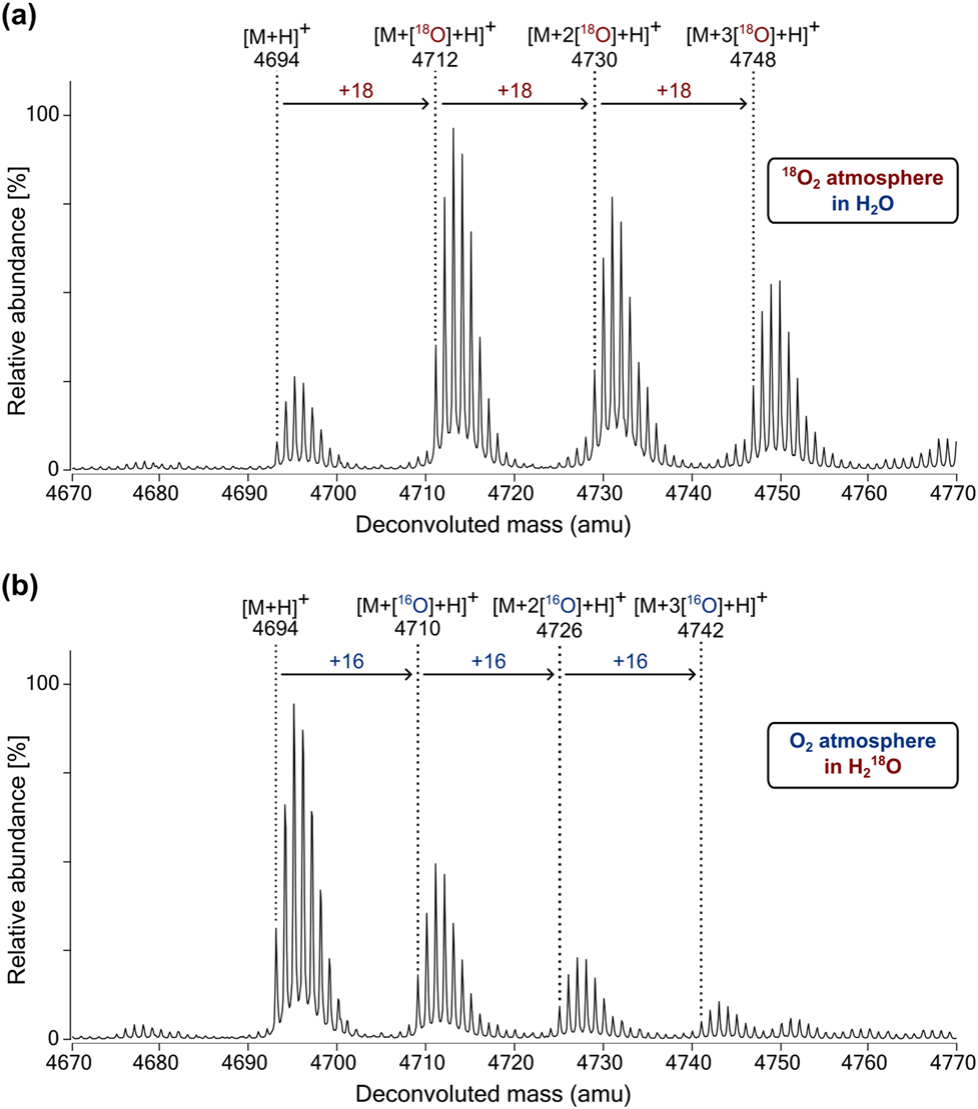
The newly incorporated oxygen atoms in hydroxylated BRD4_511–550_ originate predominantly from atmospheric O_2_. MS spectra of the JMJD6-catalysed hydroxylation of BRD4_511–550_ under (a) an ^18^O_2_ atmosphere in H_2_O, and (b) an O_2_ atmosphere in ^18^OH_2_. Assays were performed as described in the Experimental Section using full-length His_6_-JMJD6 (2 μM), 2OG (2 mM), (NH_4_)_2_Fe(SO_4_)_2_·6H_2_O (FAS; 20 μM), l-ascorbic acid (LAA; 1 mM) and BRD4_511–550_ as the substrate (50 μM) in Tris buffer (50 mM, pH 7.5).

To investigate the mechanism of oxygen incorporation into JMJD6 substrates, JMJD6-catalysed BRD4_551–550_ hydroxylation reactions were performed in the presence of ^18^O_2_ or ^18^OH_2_. Liquid chromatography-mass spectrometry (LC–MS) analyses revealed that for reactions performed under an ^18^O_2_ atmosphere, successive +18 Da shifts were observed between the BRD4_511–550_ substrate and hydroxylated products, indicating that the oxygen atom(s) incorporated into the hydroxylated products originates, at least, predominantly from ^18^O_2_ (Fig. 5a). Consistent with this proposal, products obtained from hydroxylation reactions performed in ^18^OH_2_ under an ^16^O_2_ atmosphere showed complete incorporation of ^16^O from atmospheric O_2_ (Fig. 5b). The combined results thus indicate that oxygen exchange between atmospheric O_2_ and the H_2_O is negligible during JMJD6 catalysis.

## Conclusions

There is clear evidence from genetic and animal studies that JMJD6 is a biologically important 2OG oxygenase.^38,55,57^ JMJD6 is linked to diseases and is a current medicinal chemistry target for prostate cancer.^67^ However, studies investigating its biochemical properties and the development of modulators for JMJD6 have been hampered by a lack of efficient assays measuring its catalysis. The robust SPE-MS based JMJD6 assays reported here measure the extent of JMJD6-catalysed lysyl hydroxylation in BRD-derived oligopeptides (*i.e.*, BRD2_520–559_, BRD3_463–502_ and BRD4_511–550_) and enabled efficient determination of kinetic parameters of JMJD6 for 2OG, LAA, Fe(ii), O_2_ and three BRD-derived substrate peptides. Consistent with cellular studies, under SPE-MS assay conditions, JMJD6 catalyses multiple hydroxylations of the BRD2_520–559_, BRD3_463–502_ and BRD4_511–550_ substrates. BRD2–4 are polyhydroxylated in cells, with BRD4 being apparently more extensively hydroxylated (19 hydroxylated lysine residues) than BRD3 (six hydroxylated lysine residues) and BRD2 (four hydroxylated lysine residues).^40^

Analysis of the SPE-MS derived kinetic data obtained using conditions at which mono-hydroxylation is predominant, employed for reasons of analytical simplicity, reveals that the catalytic efficiencies of BRD2_520–559_ (270 ± 110 mM^−1^ s^−1^) and BRD4_511–550_ (190 ± 80 mM^−1^ s^−1^) with JMJD6 are similar within experimental error, whilst the catalytic efficiency of BRD3_463–502_ with JMJD6 is ∼2-fold less than that of BRD4_511–550_ (87 ± 38 mM^−1^ s^−1^). Hence, the catalytic efficiencies of BRD2_520–559_, BRD3_463–502_ and BRD4_511–550_ with isolated JMJD6 are ∼200–1000-fold greater than those of reported JMJD6 peptide substrates, *e.g.*, LUC7L2_267–278_, U2AF65_30–46_ and RBM39_31–42_, which were determined using MALDI-MS based assays (Table 3).^36^ The observed substantially enhanced levels of JMJD6 catalysis under the optimised SPE-MS assay conditions with the BRD2–4-derived peptides containing multiple lysyl residues provides further support for the assignment of purified recombinant JMJD6 as a lysyl residue hydroxylase, an observation of functional relevance given that JMJD6 has also been reported to catalyse the *N*-demethylation of mono- and di-methylarginine residues,^31–35^ an activity which, however, has not been consistently reproduced subsequently.^29,36,37^

The current scope of our SPE-MS JMJD6 assays is limited to accurately determining kinetic parameters for the JMJD6-catalysed mono-hydroxylation of substrates, the kinetics of which may not necessarily represent those of subsequent hydroxylations. Note also that the catalytic efficiency of JMJD6 for full-length BRD proteins may differ from that determined with the truncated 40-mer oligopeptides employed in this work, as precedented with work on other 2OG oxygenases.^46^ Nonetheless, reported kinetic data for AspH, which, like JMJD6 (>50 human substrates^40^), is a substrate-promiscuous 2OG protein hydroxylase (>100 human substrates^48^), obtained by SPE-MS assays using EGFD-derived peptide substrates, appear to correlate, at least in some cases, with the EGFD hydroxylation levels observed in humans.^49^ This observation implies that our SPE-MS kinetic data on relative JMJD6 substrate efficiencies may, at least to some extent, be physiologically relevant.

The JMJD6-catalysed hydroxylation of BRD4_511–550_ was sensitive to low O_2_ concentrations (<2.5%), in accord with reported cellular studies.^40^ Given that the lysine-rich domain of BRD4,^68^ and other reported lysine-rich proteins including NF-κB-activating protein (NKAP),^112^ ubiquitin-specific peptidase 42 (USP42),^113^ and dyskerin pseudouridine synthase 1 (DKC1),^114^ are associated with the formation of intracellular condensates, and that JMJD6 is proposed to modulate transcriptional pause-regulation *via* its interactions with BRD4,^35^ it is possible that either or both of these processes may be regulated in an O_2_-dependent manner by JMJD6 catalysis. Our results show that the *K*^app^_m_(O_2_) value of JMJD6 (∼74 μM) is similar to that reported for the HIF-α asparagine hydroxylase FIH (∼110 μM),^28^ indicating that JMJD6 catalysis may be involved in hypoxia sensing and/or maintenance of cell homeostasis in response to limited O_2_ availability *in vivo*, as is proposed for FIH and the PHDs.^4,28,98^ It is important to note, however, that the *K*^app^_m_(O_2_) (and Fe(ii)/2OG) values do not necessarily reflect *K*_D_ values for 2OG oxygenases^27,28^ and that interpretation of JMJD6 kinetic data is complicated by its oligomeric nature,^37,79^ as is the case for some other 2OG oxygenases.^85,89^

It has been proposed an alternative measure of determining whether a 2OG oxygenase is tailored to act in an O_2_ availability sensing role by measuring the rate of reaction of O_2_ with the ternary enzyme:Fe(ii):2OG:substrate complex under single turnover conditions, with human PHD2 manifesting unusually slow O_2_ binding/reaction kinetics, consistent with its role in sensing hypoxia/O_2_ availability.^27,94^ These types of assays could be pursued in future work with JMJD6, but are complicated by the oligomeric nature of JMJD6^37,79^ and its proclivity for self-hydroxylation.^43^ It is also possible that the kinetic parameters of the reaction of O_2_ with JMJD6 (and other apparently promiscuous 2OG oxygenases) vary with different substrates (including multiple hydroxylations of the same substrate) and/or according to the cellular context. The optimised assay conditions described here will aid in investigating whether JMJD6- and O_2_-mediated poly-hydroxylation affects the fold and/or physicochemical properties of its substrates, the results of which may inform on the biological role(s) of JMJD6-catalysed substrate oxidation.

The *K*^app^_m_ value of JMJD6 for 2OG was relatively high compared to those reported for other human 2OG protein hydroxylases, including AspH,^49^ FIH,^82^ JMJD5^74^ and PHD2^88^ (Table 2). Although *K*^app^_m_ values may not reflect *K*^app^_D_ values, this result suggests relatively weak binding of 2OG by JMJD6 is a possibility, an observation which may reflect the relatively open JMJD6 active site compared to other 2OG oxygenases, as observed in JMJD6 crystal structures,^36,115,116^ and/or the oligomeric nature of JMJD6.^37,79^ Interestingly, the *K*^app^_m_ value of JMJD6 for 2OG was ∼6-fold lower than that reported for human BBOX, a dimeric oxygenase for which consistently high 2OG values have been reported.^83,84^ Nonetheless, given that the reported cellular concentration of 2OG is relatively high (∼1 mM),^86^ it might be considered unlikely that hydroxylation of BRD2–4 by JMJD6 may be regulated by 2OG availability in cells.

The addition of LAA to the assay buffer significantly enhanced levels of JMJD6 catalysis, reminiscent of the effect of LAA on catalysis of PHD2,^92,93^ FIH,^92^ ten-eleven translocation (TET) proteins,^117^ and procollagen prolyl hydroxylases.^91^ The precise mechanism through which LAA enhances JMJD6 activity is unclear; however, it has been proposed that LAA increases the activity of other 2OG oxygenases by serving as a reducing agent to maintain the active site Fe in its catalytically active Fe(ii) oxidation state.^95,118^ Fe(ii) can be oxidized to Fe(iii) *via* substrate-uncoupled turnover, which must then be reduced back to Fe(ii) to regenerate catalytically active enzyme.^94,95,118^ Fe(ii) can also undergo autooxidation in buffers.^119^ However, caution should be taken with respect to assigning a direct biologically relevant role to LAA in JMJD6 catalysis, since the enzymatic activities of some 2OG oxygenases (*e.g.*, the *Escherichia coli* DNA demethylase AlkB) can be maintained by alternative reducing agents in the absence of LAA.^120^

2OG oxygenases have emerged as important human therapeutic and agrochemical targets.^121^ Inhibitors of the PHDs are used for the treatment of anaemia caused by chronic kidney disease^122^ and the plant 2OG oxygenase gibberellin C20-oxidase is the molecular target of plant-growth retardants.^121^ Cellular studies indicate that aberrant JMJD6 activity may promote cancer development and/or progression;^64–67^ thus, selective inhibition of JMJD6 catalysis may enable the development of anti-cancer therapeutics, including for prostate cancer. The high levels of JMJD6 catalysis observed under the optimized SPE-MS assay conditions compared to *e.g.*, reported MALDI-MS JMJD6 assays (Table 3),^36^ will enable high-throughput SPE-MS based JMJD6 inhibition studies. The development of SPE-MS assays for the similarly protein substrate-promiscuous 2OG protein hydroxylases FIH and AspH as well as for the JmjC hydroxylase JMJD5, which all are also current medicinal chemistry targets, has demonstrated the potential of SPE-MS to accelerate development of potent and selective small-molecule 2OG oxygenase inhibitors.^74,123–128^ Equivalent SPE-MS JMJD6 inhibition assays would not only be valuable to support JMJD6 inhibitor development programs, but also to enable studies on the selectivity of reported 2OG oxygenase inhibitors. Given the severe phenotypes observed following JMJD6 knockout,^56–60^ this is of particular importance with respect to clinically-used inhibitors of the PHDs for long-term treatment of anaemia.

## Experimental

### General information

All chemicals were from commercial sources (Sigma-Aldrich) and were used as received. Milli-Q® ultrapure (MQ-grade) water was used for buffer preparation; LC–MS grade solvents were used for assay buffers and SPE-MS solvents. Cofactor/cosubstrate stock solutions (LAA: 50 mM in MQ-grade water; 2OG: 10 mM in MQ-grade water; ammonium iron(ii) sulfate hexahydrate, FAS, (NH_4_)_2_Fe(SO_4_)_2_·6H_2_O: 400 mM in 20 mM HCl diluted to 1 mM in MQ-grade water) were freshly prepared from commercial solids on the day of use.

### Recombinant JMJD6 production and purification

Recombinant human JMJD6 (full length with a N-terminal His_6_-tag) was produced in *Escherichia coli* BL21 (DE3) cells using a pET-28a(+) plasmid and purified by standard Ni(ii)-affinity size-exclusion chromatography, as previously reported.^40^ JMJD6 was >95% pure by SDS-PAGE and MS analysis (Fig. S1, ESI†). Purified JMJD6 was stored at −78 °C; fresh aliquots were used for all JMJD6 assays.

### JMJD6 substrates

The JMJD6 substrate peptides used for kinetic studies *i.e.*, BRD2_520–559_ (LAELQEQLRAVHEQLAALSQGPISKPKRKREKKEKKKKRK), BRD3_463–502_ (LAELQEQLKAVHEQLAALSQAPVNKPKKKKEKKEKEKKKK), and BRD4_511–550_ (LAELQEQLKAVHEQLAALSQPQQNKPKKKEKDKKEKKKEK) are based on the sequences of human BRD2, BRD3, and BRD4 and were synthesized by solid-phase peptide synthesis and were purified by GL Biochem (Shanghai) Ltd (Shanghai, China). Peptides were used as 10 mM stock solutions in DMSO (Sigma-Aldrich; BioUltra grade).

### Kinetic parameters of JMJD6 for 2OG, Fe(ii), LAA and BRD-derived substrates

Assays to investigate the kinetic parameters of JMJD6 for 2OG, Fe(ii), LAA, BRD2_520–559_, BRD3_463–502_ and BRD4_511–550_ were performed in 96-deep well polypropylene assay plates (Greiner) in independent triplicates. An enzyme mixture (10 μL) containing full-length His_6_-JMJD6 (5 μM; final concentration: 0.05 μM) in 50 mM Tris buffer (pH 7.5) was added at ambient temperature to a substrate mixture (1.0 mL) containing 2OG, Fe(ii), LAA and a BRD-derived substrate peptide (final concentrations as specified in Fig. S3–S8, ESI†). The JMJD6-catalysed hydroxylation of the BRD-derived substrate peptide was monitored at ambient temperature by SPE-MS using a RapidFire RF 365 high-throughput sampling robot (Agilent) attached to an iFunnel Agilent 6550 accurate mass quadrupole time-of-flight (Q-TOF) mass spectrometer, which was operated in the positive ionization mode. Assay samples were aspirated under vacuum for 0.4 s, then loaded onto a C4 solid phase extraction (SPE) cartridge. After loading, the C4 SPE cartridge was washed with 0.1%_v/v_ aqueous formic acid to remove non-volatile buffer salts (5 s, 1.5 mL min^−1^). The peptide was eluted from the SPE cartridge with 0.1%_v/v_ aqueous formic acid in 75/25_v/v_ acetonitrile/water into the mass spectrometer (5 s, 1.6 mL min^−1^) and the SPE cartridge re-equilibrated with 0.1%_v/v_ aqueous formic acid (1 s, 1.25 mL min^−1^).

Peaks corresponding to the *m/z* +5 charge states of the BRD-derived substrate peptide and the hydroxylated product peptides were extracted from the ion chromatogram and integrated using RapidFire Integrator 4.3.0 (Agilent). Peak area data were exported into Microsoft Excel and used to calculate the relative abundance of the mono-hydroxylated product peptide using the following equation:

% of mono-hydroxylated product peptide = 100 × (integral of mono-hydroxylated product peptide)/(integral of non-hydroxylated substrate peptide + sum of integrals of all hydroxylated product peptides). Note that for the time-period used to calculate initial reaction velocities, negligible amounts of multi-hydroxylated product peptide were observed (<5%); therefore, the sum of integrals of all hydroxylated product peptides ≈ the integral of the mono-hydroxylated product peptide. Analogous equations were used to calculate the relative abundance of the non-hydroxylated substrate peptide, the di-hydroxylated product peptide, the tri-hydroxylated product peptide (for BRD2_520–559_ and BRD3_463–502_ only) and the tetra-hydroxylated product peptide (for BRD2_520–559_ only).

The concentration of the mono-hydroxylated product peptide was calculated by multiplying the %-fraction of the mono-hydroxylated product peptide by the initial concentration of the substrate peptide employed in the assay. Initial reaction velocities were then calculated and fitted to a Michaelis–Menten plot using nonlinear regression (GraphPad Prism 5).

### Kinetic parameters of JMJD6 for O_2_

Assays to determine the kinetic parameters of JMJD6 for O_2_ were performed in 2 mL gas-tight glass vials (Sigma-Aldrich) in independent triplicates. 50 mM Tris buffer (pH 7.5; 0.95 mL) was incubated in a 2 mL gas-tight glass vial at 37 °C for 30 min with a fixed O_2_ concentration (in N_2_; specified in Fig S9, ESI†), adjusted using a mass flow controller. The substrate mixture (40 μL), containing LAA (2 mM; *i.e.*, 25 × final concentration), 2OG (2.5 mM), FAS (50 μM) and BRD4_511–550_ (100 μM) in 50 mM Tris buffer (pH 7.5), and the enzyme mixture (10 μL), containing full-length His_6_-JMJD6 (1 μM; *i.e.*, 100 × final concentration) in 50 mM Tris buffer (pH 7.5), were sequentially added to the buffer solution using a gas-tight syringe. The JMJD6-catalysed reaction was performed at 37 °C at a fixed O_2_ concentration and was stopped after the reaction time specified (Fig. S9, ESI†) by the addition of 20%_v/v_ aqueous formic acid (100 μL) and analysed by SPE-MS. The %-concentration of O_2_ in N_2_, adjusted with a mass flow controller, was converted into an O_2_ concentration [μM] by calibration using a standard (*i.e.*, *y*_[μM]_ = 9.026·*x*_[%]_).^100^ Data were analysed by SPE-MS as described above to calculate apparent JMJD6 kinetic parameters for O_2_.

### Hydroxylation assays under an ^18^O_2_ atmosphere

Assays to investigate JMJD6-catalysed hydroxylation of BRD4_511–550_ under an ^18^O_2_ atmosphere were performed as follows: assay mixtures (200 μL) containing full-length His_6_-JMJD6 (2 μM), BRD4_511–550_ (50 μM), LAA (1 mM), FAS (20 μM) and 2OG (2 mM) in 50 mM Tris (pH 7.5) were prepared in 500 μL microfuge tubes in an anaerobic chamber (N_2_ atmosphere). Cofactor and buffer solutions were prepared using degassed Milli-Q® Ultrapure grade water and JMJD6 stocks were incubated in the anaerobic chamber prior to use. The assay solutions were transferred to a custom-made air-tight container, which was then sealed and removed from the anaerobic chamber. The outlet of the container was connected to a glass manifold that was itself connected to a N_2_ line, an ^18^O_2_ cylinder and a vacuum pump. The container was evacuated and then filled with N_2_ gas to a pressure of ∼700 mbar. The container was then filled with ^18^O_2_ gas to a pressure of ∼1000 mbar, *i.e.*, to approximately mimic atmospheric oxygen concentrations. The enzyme reactions were incubated for 24 h at room temperature, after which time the container was returned to the anaerobic chamber. The reactions were stopped by the addition of 10%_v/v_ aqueous formic acid (20 μL) and analysed by LC–MS using an Agilent 1290 infinity II LC system comprising a 1290 infinity II multi-sampler and a 1290 infinity II high speed pump connected to an Agilent 6550 accurate mass iFunnel quadrupole time-of-flight (QTOF) mass spectrometer. Samples (6 μL) were injected onto a 2.1 × 50 mm, 1.8 μm ZORBAX RRHD Eclipse Plus C18 column equipped with a UHPLC guard column (Agilent; flow rate: 0.2 mL min^−1^). The mobile phase solvent A comprised 100%_v/v_ LCMS grade water containing 0.1%_v/v_ LCMS grade formic acid and mobile phase solvent B comprised 100%_v/v_ acetonitrile containing 0.1%_v/v_ LCMS grade formic acid. The peptide was separated from JMJD6 using a stepwise gradient (0 min–0%_v/v_ solvent B, 4 min–0%_v/v_ solvent B, 7 min–30%_v/v_ solvent B, 8 min–95%_v/v_ solvent B, 9 min–95%_v/v_ solvent B, 10 min–5%_v/v_ solvent B). The column was then re-equilibrated with a 1.5-min post-run with 100%_v/v_ solvent A. The mass spectrometer was operated in the positive electrospray ionization (ESI) mode with a nitrogen drying gas temperature (280 °C), drying gas flow rate (13 L min^−1^), nebulizer pressure (40 psig), sheath gas temperature (350 °C), sheath gas flow rate (12 L min^−1^), capillary voltage (4000 V), nozzle voltage (1000 V), fragmentor voltage (365 V). Acquired data were analysed using Agilent MassHunter Qualitative Analysis (version B.07.00).

### Hydroxylation assays in ^18^OH_2_

Assays to investigate the JMJD6-catalysed hydroxylation of BRD4_511–550_ in ^18^OH_2_ were performed as follows: assay mixtures (200 μL) containing full-length His_6_-JMJD6 (2 μM), BRD4_511–550_ (50 μM), LAA (1 mM), FAS (20 μM) and 2OG (2 mM) in 50 mM Tris (pH 7.5) were prepared in 500 μL microfuge tubes. Tris buffer, cofactor and BRD4_511–550_ stock solutions were prepared using ^18^OH_2_ (CK Isotopes) and JMJD6 was buffer exchanged into 50 mM Tris (pH 7.5) in ^18^OH_2_. The enzyme reactions were incubated under an ambient atmosphere at room temperature for 24 h, after which the reactions were stopped by the addition of 10%_v/v_ aqueous formic acid (20 μL) and analysed by LC–MS as described above.

## Abbreviations

BRD: Bromodomain-containing protein
FIH: Factor inhibiting hypoxia-inducible factor-α
JmjC KDM: JmjC histone *N*^ε^-methyl lysine demethylase
JMJD6: Jumonji-C domain-containing protein 6
2OG: 2-Oxoglutarate
LC–MS: Liquid chromatography–mass spectrometry
NOG: *N*-Oxalylglycine
PHD: Prolyl hydroxylase domain-containing protein
SPE-MS: Solid-phase extraction coupled to mass spectrometry.

## Data availability

The data supporting this article have been included as part of the ESI.†

## Conflicts of interest

There are no conflicts to declare.

## Supplementary Material

CB-006-D4CB00311J-s001
