# Supplementary material for: Biochemical investigations using mass spectrometry to monitor JMJD6-catalysed hydroxylation of multi-lysine containing bromodomain-derived substrates
Source: RSC Chem Biol. 2025 Feb 24;6(4):642–56. doi: 10.1039/d4cb00311j (PMC11878239; doi:10.1039/d4cb00311j)
Supplement: CB-006-D4CB00311J-s001 [file CB-006-D4CB00311J-s001.pdf]

Supplementary Information (SI) for RSC Chemical Biology.
This journal is © The Royal Society of Chemistry 2025

Supplementary Information

Biochemical investigations using mass spectrometry to monitor JMJD6-catalysed hydroxylation of multi-lysine containing bromodomain-derived substrates

Thomas P. Corner[1,+,#], Eidarus Salah[1,+], Anthony Tumber[1,+], Lennart Brewitz[1,*], and Christopher J. Schofield[1,*]

[1]Chemistry Research Laboratory, Department of Chemistry and the Ineos Oxford Institute for Antimicrobial Research, University of Oxford, 12 Mansfield Road, OX1 3TA, Oxford, United Kingdom.

*Email: lennart.brewitz@chem.ox.ac.uk, christopher.schofield@chem.ox.ac.uk

[+]These authors contributed equally to this work.

[#]Present Address: Department of Molecular, Cellular, and Developmental Biology, Yale University, New Haven, Connecticut 06511, United States of America.

Table of contents

1. Abbreviations 2 - 3
2. Supplementary figures 4 - 19
3. References 20 - 21

1. Abbreviations

2OG	2-oxoglutarate
AR	androgen receptor
AR-V7	androgen receptor slice variant 7
AspH	aspartate/asparagine β-hydroxylase
BBOX	γ-butyobetaine hydroxylase
BID	basic residue enriched interaction domain
BRD	bromodomain-containing protein
CP4H	collagen prolyl 4-hydroxylase
CROP	cisplatin resistance-associated overexpressed protein
CTAD	C-terminal activation domain
DHX9	DExH-box helicase 9
DKC1	dyskerin pseudouridine synthase 1
DSBH	double stranded β-helix
EGFD	epidermal growth factor-like domain
ERα	estrogen receptor-α
FECH	ferrochelatase
FIH	factor inducible hypoxia inducible factor-α
FLT1	vascular endothelial growth factor receptor 1
GBB	γ-butyobetaine
H3/4	histone 3/4
HIF	hypoxia-inducible factor
HSP70	heat shock protein 70
JMJD6	Jumonji-C domain-containing protein 6
KDM	histone N^{ε}-methyl lysine demethylase
K_{m}^{app}	apparent Michaelis constant
k_{cat}^{app}	turnover number
LAA	*L*-ascorbic acid
LC-MS	liquid chromatography-mass spectrometry
LUC7L2	LUC7-like 2
MALDI-MS	matrix-assisted laser desorption/ionization mass spectrometry
MINA53	MYC-induced nuclear antigen
mRNA	messenger ribonucleic acid

NKAP	NF-κB-activating protein
NMR	nuclear magnetic resonance
NOG	*N*-oxalylglycine
NO66	nucleolar protein 66
PHYH	phytanoyl-CoA dioxygenase
RBM39	RNA-binding protein 39
RPL8	ribosomal protein L8
RPL27A	ribosomal protein L27A
RPS6	ribosomal protein S6
O_2	dioxygen
PDB	protein data bank
PHD	prolyl hydroxylase domain-containing protein
pVHL	von Hippel-Lindau protein
SRSF11	serine and arginine rich splicing factor 11
SPE-MS	solid-phase extraction coupled to mass spectrometry
TET	ten-eleven translocation
TRAF6	tumor necrosis factor receptor associated factor 6
U2AF65	splicing factor U2 auxiliary factor 65 kDa subunit
U2SURP	U2 SnRNP associated SURP domain containing
USP42	ubiquitin-specific peptidase 42
v_{max}^{app}	apparent maximum velocities

2. Supplementary figures

Supplementary Figure S1. Recombinant human JMJD6. Recombinant human JMJD6 (full-length with an N-terminal His_6-tag) was >95% pure as analysed by sodium dodecyl sulphate polyacrylamide gel electrophoresis (SDS-PAGE) with Coomassie staining and electrospray ionization mass spectrometry (ESI-MS).

(**a**) 10-12% SDS-PAGE analysis of purified His_6-JMJD6 after Ni(II)-affinity chromatography (HisTrap column); (**b**) 10-12% SDS-PAGE analysis of His_6-JMJD6 after subsequent size-exclusion chromatography (Superdex 75); (**c**) Deconvoluted mass spectrum of purified His_6-JMJD6 shows potential evidence for JMJD6 self-hydroxylation, as reported;[1] calculated mass of His_6-JMJD6 = 49014.73 Da, observed mass: 49030.24 Da (*i.e.*, mass difference: ~ +16 Da).

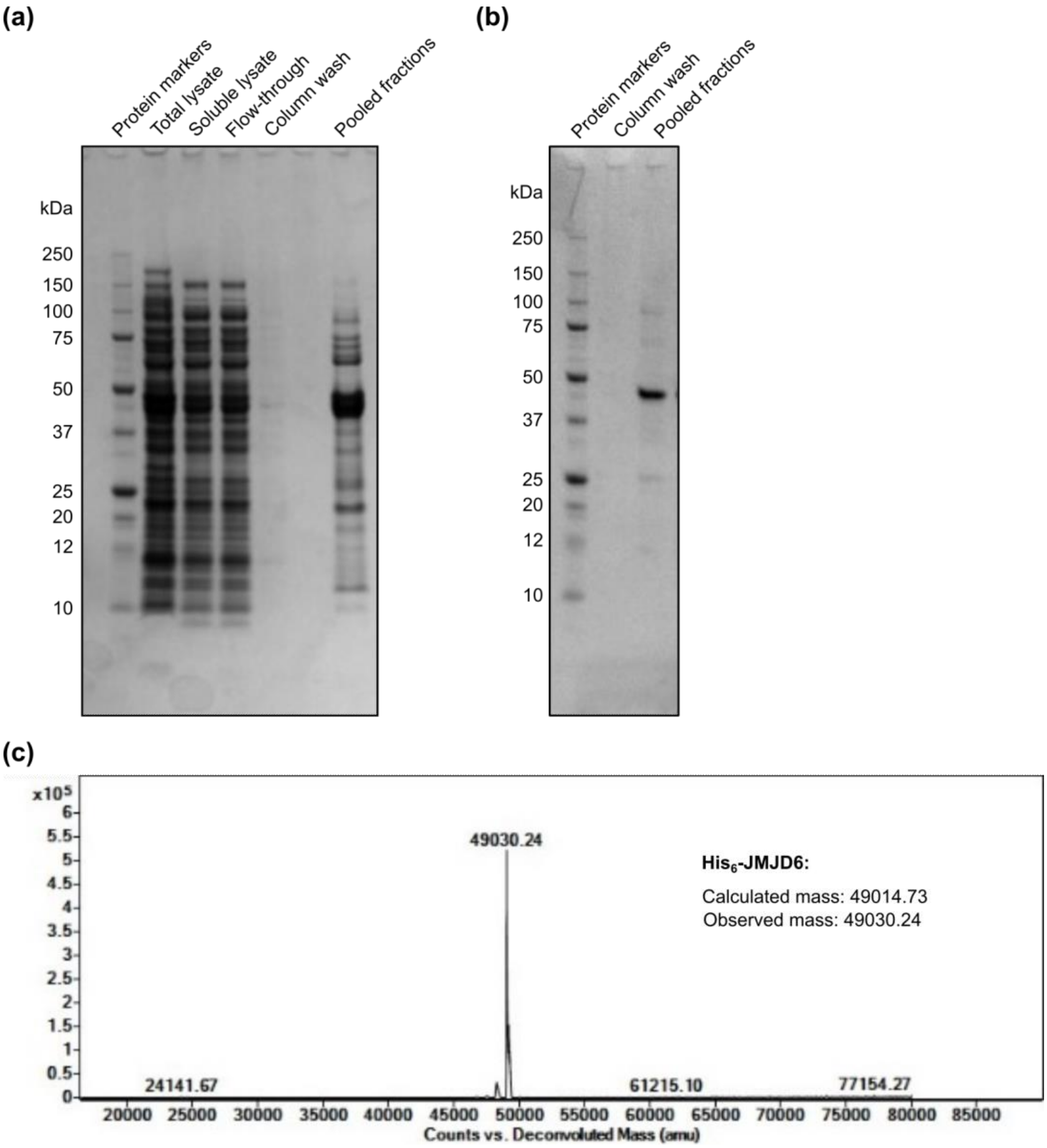

Supplementary Figure S2. Ion counts of BRD-derived peptides measured during JMJD6-catalysed hydroxylation. The sum of the MS ion counts of the substrate peptide and all hydroxylated product peptides (black line) remains approximately constant throughout the JMJD6-catalyzed reactions of: (**a**) $BRD2_{520-559}$, (**b**) $BRD3_{463-502}$ and (**c**) $BRD4_{511-550}$. The results indicate that the SPE-MS assays are sufficiently robust to enable quantification of the extent of the JMJD6-catalysed $BRD2_{520-559}$, $BRD3_{463-502}$ and $BRD4_{511-550}$ hydroxylation. Hydroxylation reactions were performed as described in the Experimental Procedures section using His_6-JMJD6 (0.05 µM), 2OG (200 µM), $(NH_4)_2Fe(SO_4)_2 \cdot 6H_2O$ (FAS; 2 µM), BRD substrate (2 µM) and *L*-ascorbic acid (LAA; 100 µM) in Tris buffer (50 mM, pH 7.5).

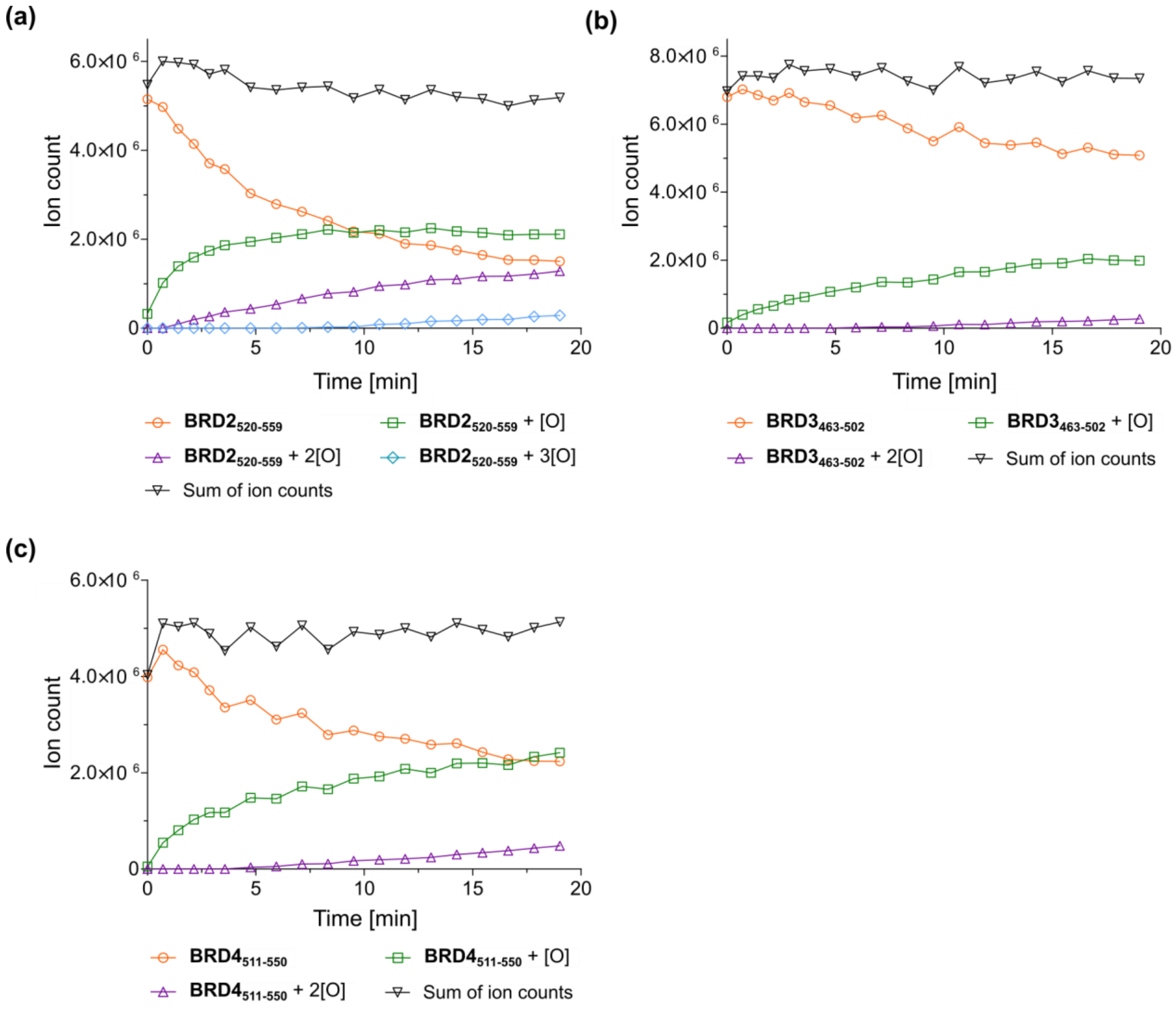

Supplementary Figure S3. Determination of the JMJD6 kinetic parameters for 2OG (continues on the following page). SPE-MS was used to determine the extent of JMJD6-catalysed mono- and di-hydroxylation of the $BRD4_{511\text{-}550}$ fragment peptide for the specified 2OG concentrations. Initial rates of the JMJD6-catalysed mono-hydroxylation of $BRD4_{511\text{-}550}$ were used to determine the maximum velocity (v_{max}^{app}) and Michaelis constant (K_m^{app}) of JMJD6 for 2OG. Measurement times were normalized to the first sample injection analysed after the addition of the JMJD6 enzyme mixture to the substrate mixture (containing 2OG, FAS, LAA, and $BRD4_{511\text{-}550}$), by which time low levels of $BRD4_{511\text{-}550}$ mono-hydroxylation were manifest. Data are the mean of three independent runs (n = 3; mean ± standard deviation, SD). Conditions: His_6-JMJD6 (50 nM), 2OG (as specified), FAS (2 µM), $BRD4_{511\text{-}550}$ (2 µM) and LAA (100 µM) in Tris buffer (50 mM, pH 7.5).

(**a**) Abundance of the mono-hydroxylated $BRD4_{511\text{-}550}$ peptide, following the addition of 50 nM JMJD6 to the substrate mixture (t = 0 min), for the specified 2OG concentrations; (**b**) initial reaction velocities used for the kinetic analysis of the JMJD6-catalysed mono-hydroxylation of $BRD4_{511\text{-}550}$; (**c**) Michaelis-Menten curve used to determine the kinetic parameters of JMJD6 for 2OG. The JMJD6 v_{max}^{app} and K_m^{app} values of JMJD6 for 2OG are $3.5 \pm 0.1 \cdot 10^{-3}$ $\mu M \cdot s^{-1}$ and 23.3 ± 2.5 µM, respectively, as determined by non-linear regression; (**d**-**l**) time course of the JMJD6-catalysed hydroxylation of $BRD4_{511\text{-}550}$ showing the relative abundance of the $BRD4_{511\text{-}550}$ substrate ($BRD4_{511\text{-}550}$; orange circles), mono-hydroxylated $BRD4_{511\text{-}550}$ ($BRD4_{511\text{-}550}$ + [O]; green squares) and di-hydroxylated $BRD4_{511\text{-}550}$ ($BRD4_{511\text{-}550}$ + 2[O]; purple triangles), in the presence of varied 2OG concentrations: (**d**) 400 µM; (**e**) 200 µM; (**f**) 100 µM; (**g**) 50 µM; (**h**) 25 µM; (**i**) 12.5 µM; (**j**) 6.25 µM; (**k**) 3.13 µM; (**l**) 0 µM. No evidence for triple (or higher order) $BRD4_{511\text{-}550}$ hydroxylation was observed under the employed reaction conditions.

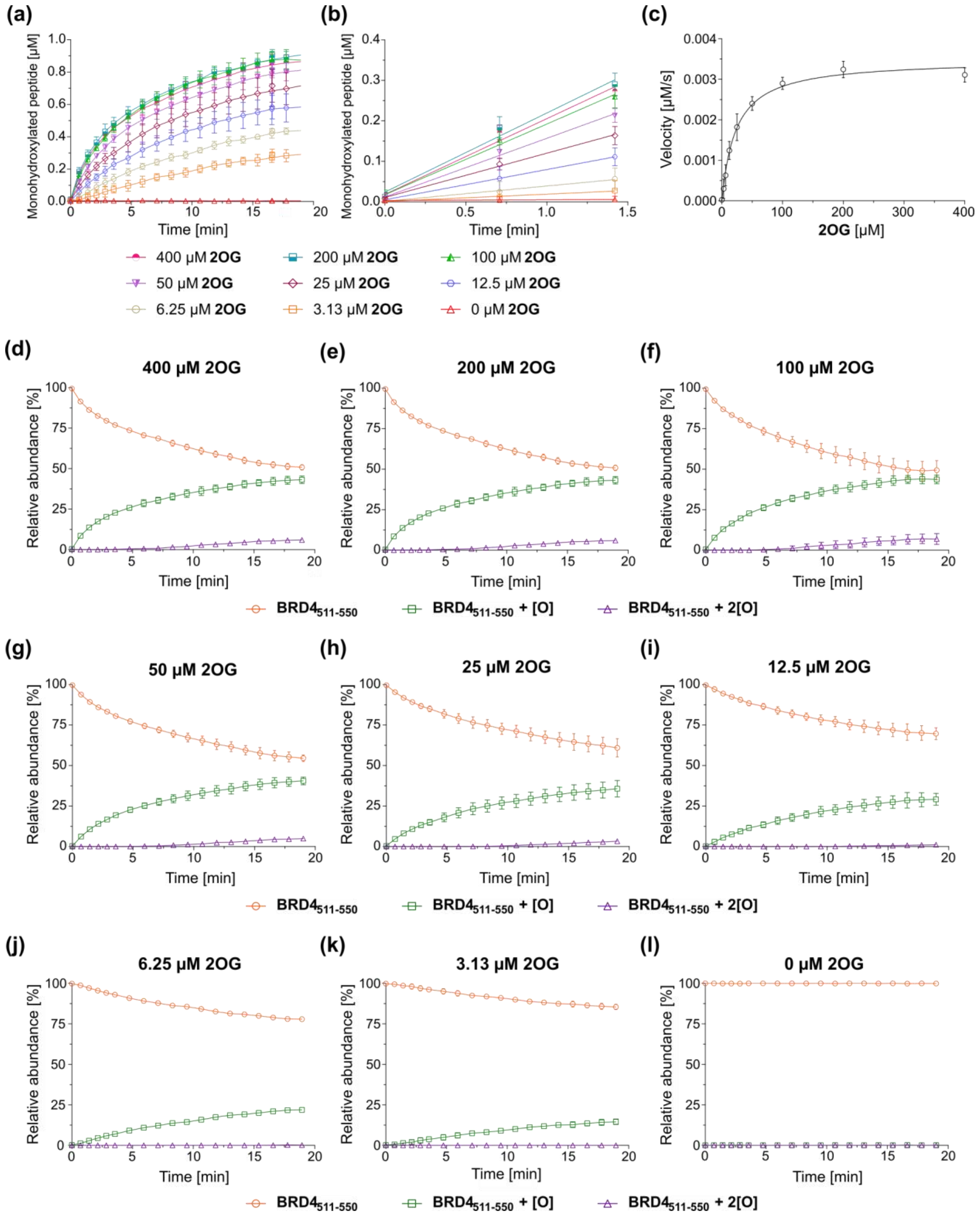
(a)
Monohydroxylated peptide [µM]
Time [min]
(b)
Monohydroxylated peptide [µM]
Time [min]
(c)
Velocity [µM/s]
2OG [µM]
400 µM 2OG
200 µM 2OG
100 µM 2OG
50 µM 2OG
25 µM 2OG
12.5 µM 2OG
6.25 µM 2OG
3.13 µM 2OG
0 µM 2OG
(d) 400 µM 2OG
(e) 200 µM 2OG
(f) 100 µM 2OG
(g) 50 µM 2OG
(h) 25 µM 2OG
(i) 12.5 µM 2OG
(j) 6.25 µM 2OG
(k) 3.13 µM 2OG
(l) 0 µM 2OG
Relative abundance [%]
Time [min]
BRD4511-550
BRD4511-550 + [O]
BRD4511-550 + 2[O]

Supplementary Figure S4. Determination of the JMJD6 kinetic parameters for Fe(II) (continues on the following page). SPE-MS was used to determine the extent of JMJD6-catalysed mono- and di-hydroxylation of the $BRD4_{511\text{-}550}$ fragment peptide for the specified Fe(II) concentrations. Initial rates of the JMJD6-catalysed mono-hydroxylation of $BRD4_{511\text{-}550}$ were used to determine the maximum velocity (v_{max}^{app}) and apparent Michaelis constant (K_m^{app}) of JMJD6 for Fe(II). Measurement times were normalized to the first sample injection analysed after the addition of the JMJD6 enzyme mixture into the substrate mixture, by which time low levels of $BRD4_{511\text{-}550}$ mono-hydroxylation were manifest. Data are the mean of three independent runs (n = 3; mean ± SD). Conditions: His_6-JMJD6 (50 nM), 2OG (200 µM), FAS (as specified), $BRD4_{511\text{-}550}$ (2 µM) and LAA (100 µM) in Tris buffer (50 mM, pH 7.5).

(**a**) Abundance of the mono-hydroxylated $BRD4_{511\text{-}550}$ peptide, following the addition of 50 nM JMJD6 to the substrate mixture (t = 0 min), for the specified Fe(II) concentrations; (**b**) initial reaction velocities used for the kinetic analysis of the JMJD6-catalysed mono-hydroxylation of $BRD4_{511\text{-}550}$; (**c**) Michaelis-Menten curve used to determine the kinetic parameters of JMJD6 for Fe(II). The JMJD6 v_{max}^{app} and K_m^{app} values of JMJD6 for Fe(II) are $3.6 \pm 0.1 \cdot 10^{-3}$ $\mu M \cdot s^{-1}$ and 0.19 ± 0.02 µM, respectively, as determined by non-linear regression; (**d**-**k**) time course of the JMJD6-catalysed hydroxylation of $BRD4_{511\text{-}550}$ showing the relative abundance of the $BRD4_{511\text{-}550}$ substrate ($BRD4_{511\text{-}550}$; orange circles), mono-hydroxylated $BRD4_{511\text{-}550}$ ($BRD4_{511\text{-}550}$ + [O]; green squares) and di-hydroxylated $BRD4_{511\text{-}550}$ ($BRD4_{511\text{-}550}$ + 2[O]; purple triangles), in the presence of varied Fe(II) concentrations: (**d**) 4 µM; (**e**) 2 µM; (**f**) 1 µM; (**g**) 0.5 µM; (**h**) 0.25 µM; (**i**) 0.125 µM; (**j**) 0.063 µM; (**k**) 0 µM. No evidence for triple (or higher order) $BRD4_{511\text{-}550}$ hydroxylation was observed under the employed reaction conditions.

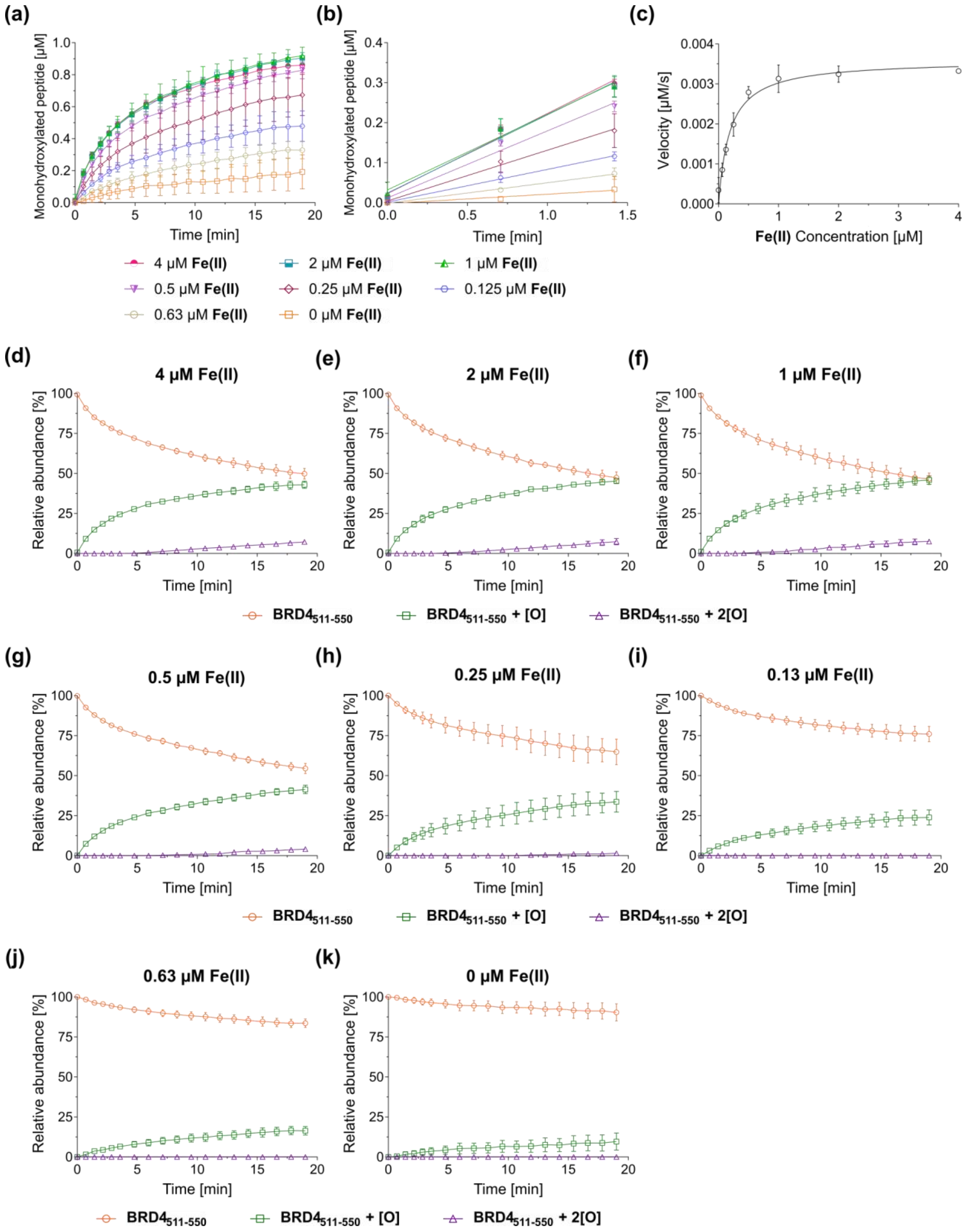
(a)
(b)
(c)
Monohydroxylated peptide [µM]
Time [min]
Velocity [µM/s]
Fe(II) Concentration [µM]
4 µM Fe(II)
2 µM Fe(II)
1 µM Fe(II)
0.5 µM Fe(II)
0.25 µM Fe(II)
0.125 µM Fe(II)
0.63 µM Fe(II)
0 µM Fe(II)
(d) 4 µM Fe(II)
(e) 2 µM Fe(II)
(f) 1 µM Fe(II)
(g) 0.5 µM Fe(II)
(h) 0.25 µM Fe(II)
(i) 0.13 µM Fe(II)
(j) 0.63 µM Fe(II)
(k) 0 µM Fe(II)
Relative abundance [%]
Time [min]
BRD4511-550
BRD4511-550 + [O]
BRD4511-550 + 2[O]

Supplementary Figure S5. Effect of LAA on the JMJD6-catalysed hydroxylation of $BRD4_{511\text{-}550}$ (continues on the following page). SPE-MS was used to determine the extent of JMJD6-catalysed mono- and di-hydroxylation of the $BRD4_{511\text{-}550}$ fragment peptide for the specified concentrations of L-ascorbic acid (LAA). Measurement times were normalized to the first sample injection analysed after the addition of the JMJD6 enzyme mixture to the substrate mixture, by which time low levels of $BRD4_{511\text{-}550}$ mono-hydroxylation were manifest. Data are the mean of three independent runs (n = 3; mean ± SD). Conditions: His_6-JMJD6 (50 nM), 2OG (200 µM), FAS (2 µM), $BRD4_{511\text{-}550}$ (2 µM) and LAA (as specified) in Tris buffer (50 mM, pH 7.5).

(**a**) Abundance of the mono-hydroxylated $BRD4_{511\text{-}550}$ peptide, following the addition of 50 nM JMJD6 to the substrate mixture (t = 0 min), for the specified LAA concentrations; (**b**) initial reaction velocities used for the kinetic analysis of the JMJD6-catalysed mono-hydroxylation of $BRD4_{511\text{-}550}$; (**c**) effect of LAA concentration on the initial reaction velocity of the JMJD6-catalysed mono-hydroxylation of $BRD4_{511\text{-}550}$; (**d**-**k**) time course of the JMJD6-catalysed hydroxylation of $BRD4_{511\text{-}550}$ showing the relative abundance of the $BRD4_{511\text{-}550}$ substrate ($BRD4_{511\text{-}550}$; orange circles), mono-hydroxylated $BRD4_{511\text{-}550}$ ($BRD4_{511\text{-}550}$ + [O]; green squares) and di-hydroxylated $BRD4_{511\text{-}550}$ ($BRD4_{511\text{-}550}$ + 2[O]; purple triangles), in the presence of varied LAA concentrations: (**d**) 100 µM; (**e**) 50 µM; (**f**) 25 µM; (**g**) 12.5 µM; (**h**) 6.25 µM; (**i**) 3.13 µM; (**j**) 1.56 µM; (**k**) 0.78 µM; (**l**) 0.39 µM; (**m**) 0 µM. No evidence for triple (or higher order) $BRD4_{511\text{-}550}$ hydroxylation was observed under the employed reaction conditions.

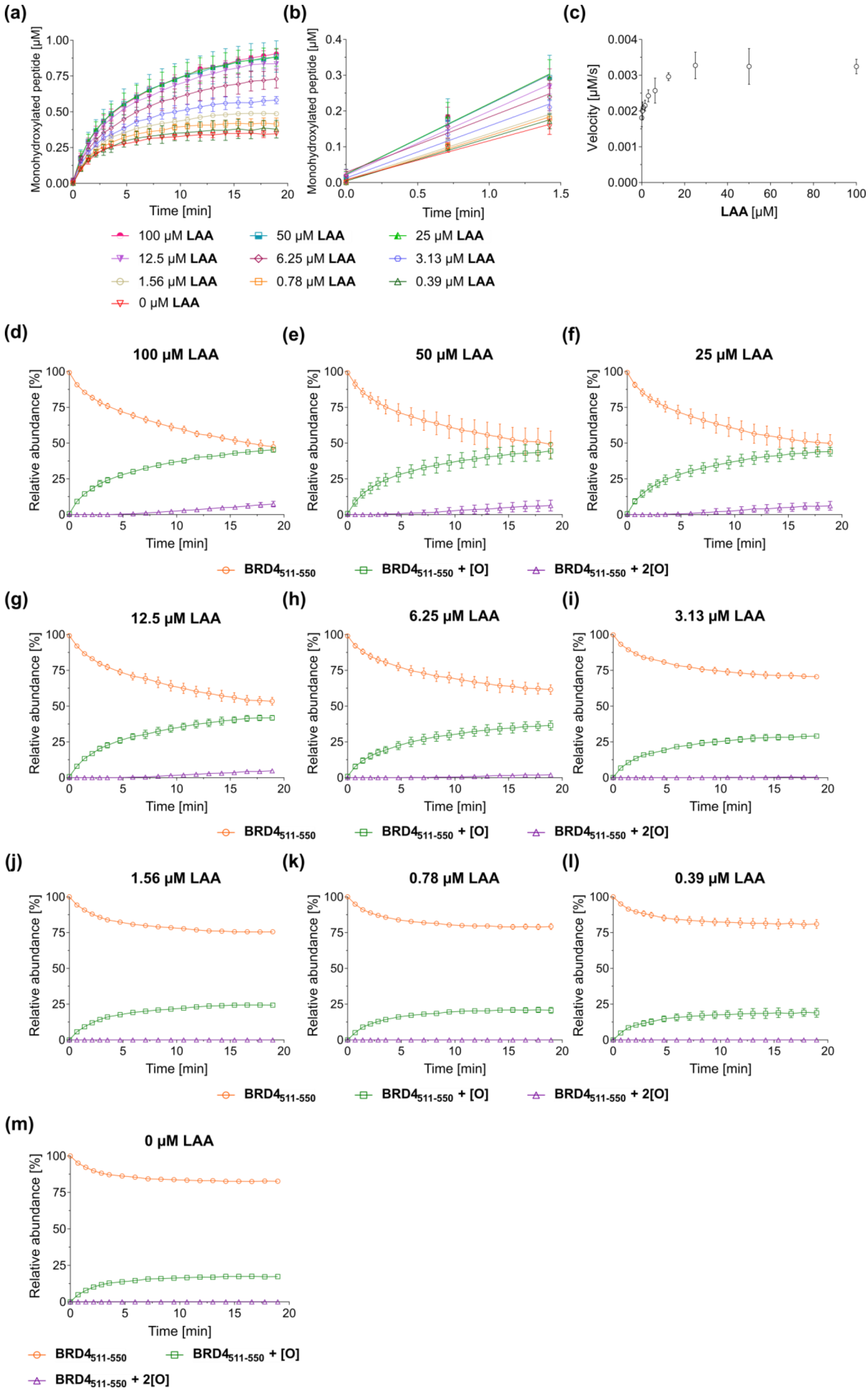

(a)
(b)
(c)
Monohydroxylated peptide [µM]
Time [min]
Velocity [µM/s]
LAA [µM]
100 µM LAA
50 µM LAA
25 µM LAA
12.5 µM LAA
6.25 µM LAA
3.13 µM LAA
1.56 µM LAA
0.78 µM LAA
0.39 µM LAA
0 µM LAA
(d)
(e)
(f)
(g)
(h)
(i)
(j)
(k)
(l)
(m)
Relative abundance [%]
BRD4$_{511-550}$
BRD4$_{511-550}$ + [O]
BRD4$_{511-550}$ + 2[O]

Supplementary Figure S6. Determination of the JMJD6 kinetic parameters for BRD4$_{511\text{-}550}$ (continues on the following page). SPE-MS was used to determine the extent of JMJD6-catalysed mono- and di-hydroxylation of the BRD4$_{511\text{-}550}$ fragment peptide for the specified BRD4$_{511\text{-}550}$ concentrations. Initial rates of the JMJD6-catalysed mono-hydroxylation of BRD4$_{511\text{-}550}$ were used to determine the maximum velocity (v_{max}^{app}) and Michaelis constant (K_m^{app}) of JMJD6 for BRD4$_{511\text{-}550}$. Measurement times were normalized to the first sample injection analysed after the addition of the JMJD6 enzyme mixture to the substrate mixture, by which time low levels of BRD4$_{511\text{-}550}$ mono-hydroxylation were manifest. Data are the mean of three independent runs (n = 3; mean ± SD). Conditions: His$_6$-JMJD6 (50 nM), 2OG (200 µM), FAS (2 µM), BRD4$_{511\text{-}550}$ (as specified) and LAA (100 µM) in Tris buffer (50 mM, pH 7.5).

(**a**) Abundance of the mono-hydroxylated BRD4$_{511\text{-}550}$ peptide, following the addition of 50 nM JMJD6 to the substrate mixture (t = 0 min), for the specified BRD4$_{511\text{-}550}$ concentrations; (**b**) initial reaction velocities used for the kinetic analysis of the JMJD6-catalysed mono-hydroxylation of BRD4$_{511\text{-}550}$; (**c**) Michaelis-Menten curve used to determine the kinetic parameters of JMJD6 for BRD4$_{511\text{-}550}$. The JMJD6 v_{max}^{app} and K_m^{app} values of JMJD6 for BRD4$_{511\text{-}550}$ are $5.8 \pm 1.2 \cdot 10^{-3}$ µM·s^{-1} and 0.62 ± 0.26 µM, respectively, as determined by non-linear regression; (**d-l**) time course of the JMJD6-catalysed hydroxylation of BRD4$_{511\text{-}550}$ showing the relative abundance of the BRD4$_{511\text{-}550}$ substrate (BRD4$_{511\text{-}550}$; orange circles), mono-hydroxylated BRD4$_{511\text{-}550}$ (BRD4$_{511\text{-}550}$ + [O]; green squares) and di-hydroxylated BRD4$_{511\text{-}550}$ (BRD4$_{511\text{-}550}$ + 2[O]; purple triangles) in the presence of varied BRD4$_{511\text{-}550}$ concentrations: (**d**) 10 µM; (**e**) 8 µM; (**f**) 6 µM; (**g**) 4 µM; (**h**) 2 µM; (**i**) 1 µM; (**j**) 0.8 µM; (**k**) 0.6 µM; (**l**) 0.4 µM. No evidence for triple (or higher order) BRD4$_{511\text{-}550}$ hydroxylation was observed under the employed reaction conditions.

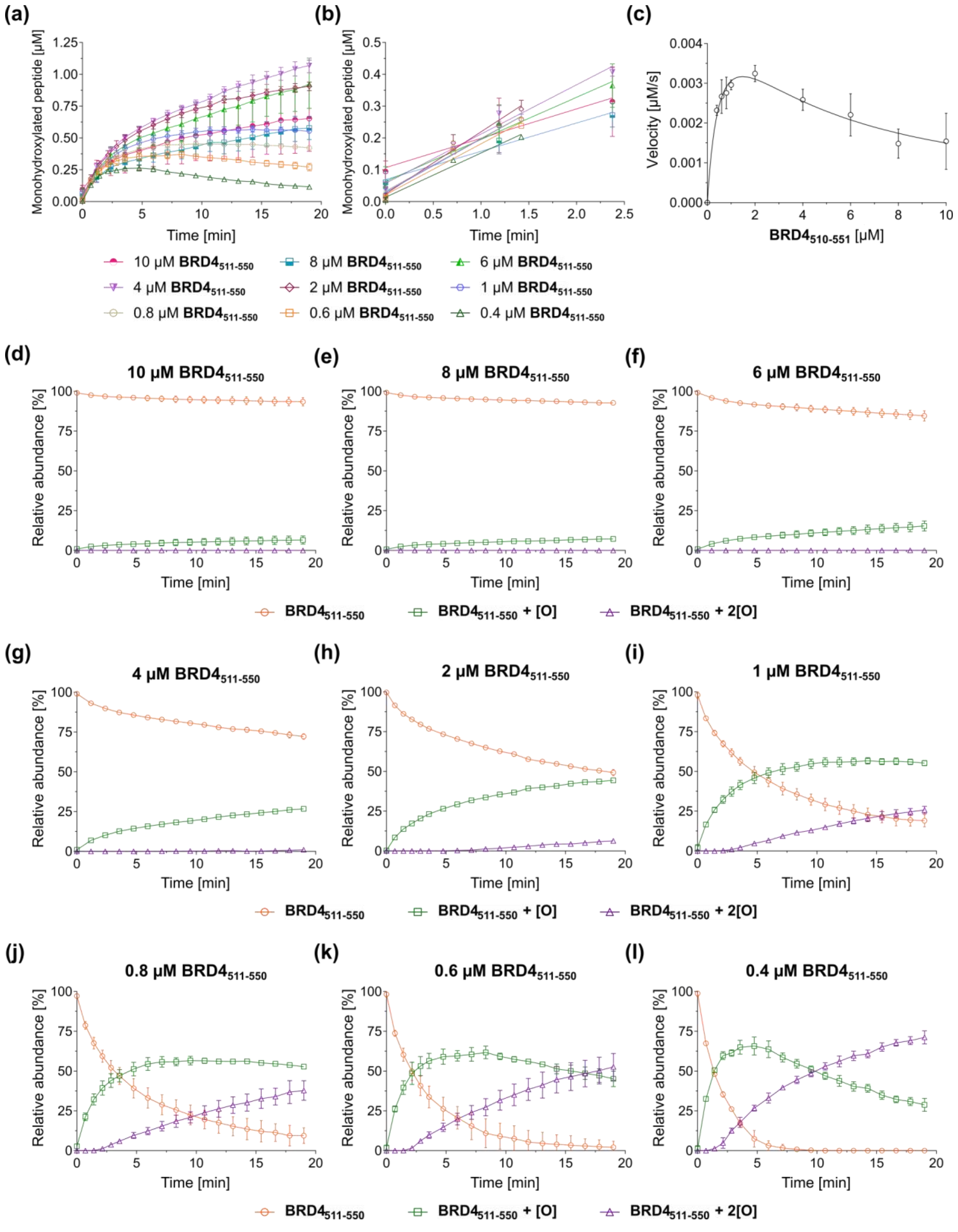
(a)
(b)
(c)
Monohydroxylated peptide [µM]
Time [min]
Velocity [µM/s]
BRD4510-551 [µM]
10 µM BRD4511-550
8 µM BRD4511-550
6 µM BRD4511-550
4 µM BRD4511-550
2 µM BRD4511-550
1 µM BRD4511-550
0.8 µM BRD4511-550
0.6 µM BRD4511-550
0.4 µM BRD4511-550
(d)
(e)
(f)
(g)
(h)
(i)
(j)
(k)
(l)
Relative abundance [%]
BRD4511-550
BRD4511-550 + [O]
BRD4511-550 + 2[O]

Supplementary Figure S7. Determination of the JMJD6 kinetic parameters for $BRD2_{520-559}$ (continues on the following page). SPE-MS was used to determine the extent of JMJD6-catalysed mono- and di-hydroxylation of the $BRD2_{520-559}$ fragment peptide for the specified $BRD2_{520-559}$ concentrations. Initial rates of the JMJD6-catalysed mono-hydroxylation of $BRD2_{520-559}$ were used to determine the maximum velocity (v_{max}^{app}) and Michaelis constant (K_m^{app}) of JMJD6 for $BRD2_{520-559}$. Measurement times were normalized to the first sample injection analysed after addition of the JMJD6 enzyme mixture to the substrate mixture, by which time low levels of $BRD2_{520-559}$ mono-hydroxylation were manifest. Data are the mean of three independent runs (n = 3; mean ± SD). Conditions: His_6-JMJD6 (50 nM), 2OG (200 μM), FAS (2 μM), $BRD2_{520-559}$ (as specified) and LAA (100 μM) in Tris buffer (50 mM, pH 7.5).

(**a**) Abundance of the mono-hydroxylated $BRD2_{520-559}$ peptide, following the addition of 50 nM JMJD6 to the substrate mixture (t = 0 min), for the specified $BRD2_{520-559}$ concentrations; (**b**) initial reaction velocities used for the kinetic analysis of the JMJD6-catalysed mono-hydroxylation of $BRD2_{520-559}$; (**c**) Michaelis-Menten curve used to determine the kinetic parameters of JMJD6 for $BRD2_{520-559}$. The JMJD6 v_{max}^{app} and K_m^{app} values of JMJD6 for $BRD2_{520-559}$ are 1.2 ± $0.2\cdot10^{-2}$ $\mu M\cdot s^{-1}$ and 0.89 ± 0.32 μM, respectively, as determined by non-linear regression; (**d-m**) time course of the JMJD6-catalysed hydroxylation of $BRD2_{520-559}$ showing the relative abundance of the $BRD2_{520-559}$ substrate ($BRD2_{520-559}$; orange circles), mono-hydroxylated $BRD2_{520-559}$ ($BRD2_{520-559}$ + [O]; green squares), di-hydroxylated $BRD2_{520-559}$ ($BRD2_{520-559}$ + 2[O]; purple triangles), trihydroxylated $BRD2_{520-559}$ ($BRD2_{520-559}$ + 3[O]; light blue diamonds) and tetrahydroxylated $BRD2_{520-559}$ ($BRD2_{520-559}$ + 4[O]; brown inverse triangles) in the presence of varied $BRD2_{520-559}$ concentrations: (**d**) 10 μM; (**e**) 8 μM; (**f**) 6 μM; (**g**) 4 μM; (**h**) 2 μM; (**i**) 1 μM; (**j**) 0.8 μM; (**k**) 0.6 μM; (**l**) 0.4 μM.

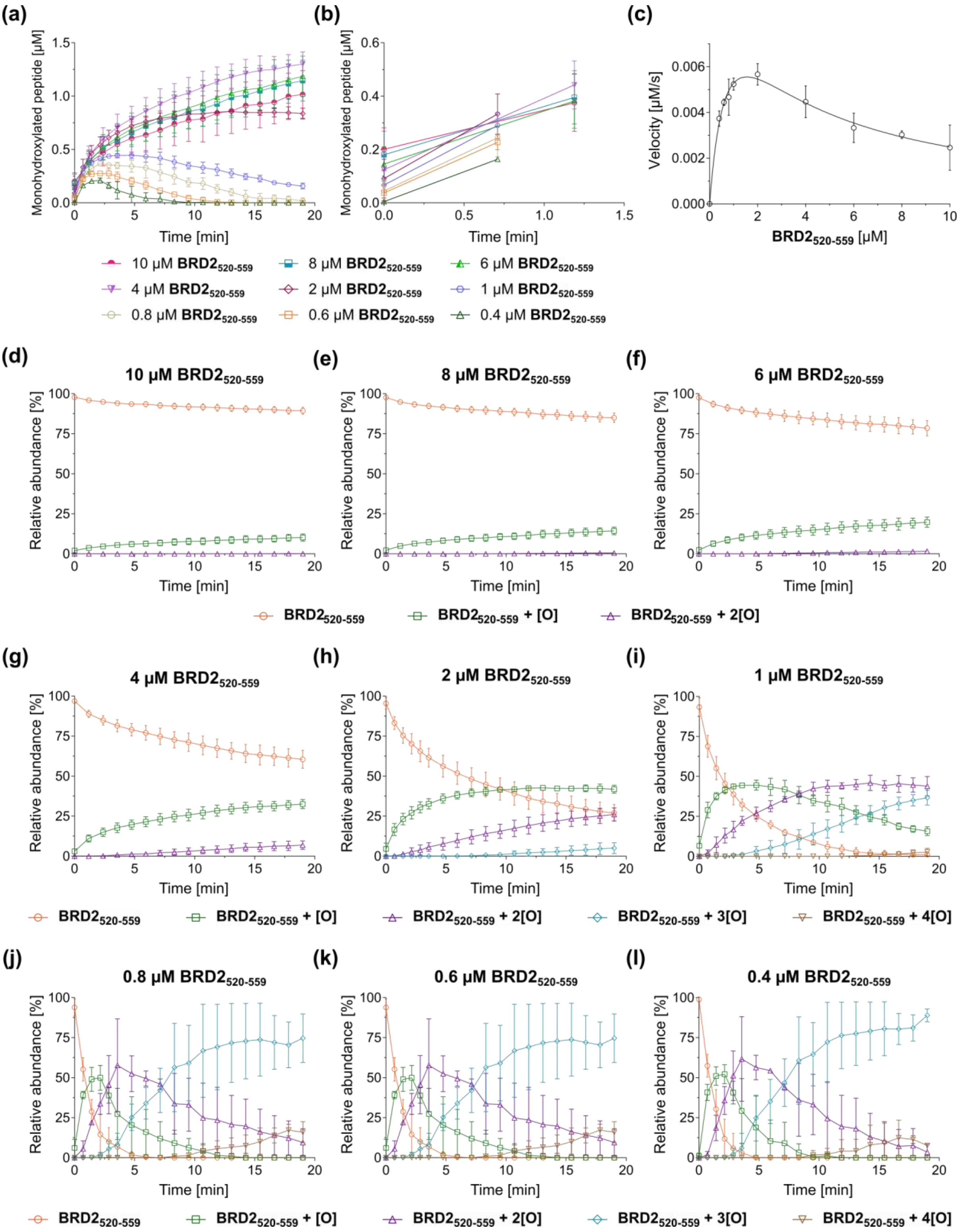
(a)
Monohydroxylated peptide [µM]
Time [min]
(b)
Monohydroxylated peptide [µM]
Time [min]
(c)
Velocity [µM/s]
BRD2$_{520-559}$ [µM]
10 µM BRD2$_{520-559}$
8 µM BRD2$_{520-559}$
6 µM BRD2$_{520-559}$
4 µM BRD2$_{520-559}$
2 µM BRD2$_{520-559}$
1 µM BRD2$_{520-559}$
0.8 µM BRD2$_{520-559}$
0.6 µM BRD2$_{520-559}$
0.4 µM BRD2$_{520-559}$
(d)
10 µM BRD2$_{520-559}$
(e)
8 µM BRD2$_{520-559}$
(f)
6 µM BRD2$_{520-559}$
Relative abundance [%]
Time [min]
BRD2$_{520-559}$
BRD2$_{520-559}$ + [O]
BRD2$_{520-559}$ + 2[O]
(g)
4 µM BRD2$_{520-559}$
(h)
2 µM BRD2$_{520-559}$
(i)
1 µM BRD2$_{520-559}$
Relative abundance [%]
Time [min]
BRD2$_{520-559}$
BRD2$_{520-559}$ + [O]
BRD2$_{520-559}$ + 2[O]
BRD2$_{520-559}$ + 3[O]
BRD2$_{520-559}$ + 4[O]
(j)
0.8 µM BRD2$_{520-559}$
(k)
0.6 µM BRD2$_{520-559}$
(l)
0.4 µM BRD2$_{520-559}$
Relative abundance [%]
Time [min]
BRD2$_{520-559}$
BRD2$_{520-559}$ + [O]
BRD2$_{520-559}$ + 2[O]
BRD2$_{520-559}$ + 3[O]
BRD2$_{520-559}$ + 4[O]

Supplementary Figure S8. Determination of the JMJD6 kinetic parameters for $BRD3_{463-502}$ (continues on the following page). SPE-MS was used to determine the extent of JMJD6-catalysed mono- and di-hydroxylation of the $BRD3_{463-502}$ fragment peptide for the specified $BRD3_{463-502}$ concentrations. Initial rates of the JMJD6-catalysed mono-hydroxylation of $BRD3_{463-502}$ were used to determine the maximum velocity (v_{max}^{app}) and Michaelis constant (K_m^{app}) of JMJD6 for $BRD3_{463-502}$. Measurement times were normalized to the first sample injection analysed after the addition of the JMJD6 enzyme mixture to the substrate mixture, by which time low levels of $BRD3_{463-502}$ mono-hydroxylation were manifest. Data are the mean of three independent runs (n = 3; mean ± SD). Conditions: His_6-JMJD6 (50 nM), 2OG (200 µM), FAS (2 µM), $BRD3_{463-502}$ (as specified) and LAA (100 µM) in Tris buffer (50 mM, pH 7.5).

(**a**) Abundance of the mono-hydroxylated $BRD3_{463-502}$ peptide, following the addition of 50 nM JMJD6 to the substrate mixture (t = 0 min), for the specified $BRD3_{463-502}$ concentrations; (**b**) initial reaction velocities used for the kinetic analysis of the JMJD6-catalysed mono-hydroxylation of $BRD3_{463-502}$; (**c**) Michaelis-Menten curve used to determine the kinetic parameters of JMJD6 for $BRD3_{463-502}$. The JMJD6 v_{max}^{app} and K_m^{app} values of JMJD6 for $BRD3_{463-502}$ are $5.2 \pm 1.3 \cdot 10^{-3}$ $\mu M \cdot s^{-1}$ and 1.2 ± 0.4 µM, respectively, as determined by non-linear regression; (**d-m**) time course of the JMJD6-catalysed hydroxylation of $BRD3_{463-502}$ showing the relative abundance of the $BRD3_{463-502}$ substrate ($BRD3_{463-502}$; orange circles), mono-hydroxylated $BRD3_{463-502}$ ($BRD3_{463-502}$ + [O]; green squares), di-hydroxylated $BRD3_{463-502}$ ($BRD3_{463-502}$ + 2[O]; purple triangles) and tri-hydroxylated $BRD3_{463-502}$ ($BRD3_{463-502}$ + 3[O]; light blue diamonds) in the presence of varied $BRD3_{463-502}$ concentrations: (**d**) 10 µM; (**e**) 8 µM; (**f**) 6 µM; (**g**) 4 µM; (**h**) 2 µM; (**i**) 1 µM; (**j**) 0.8 µM; (**k**) 0.6 µM; (**l**) 0.4 µM; (**m**) 0.2 µM.

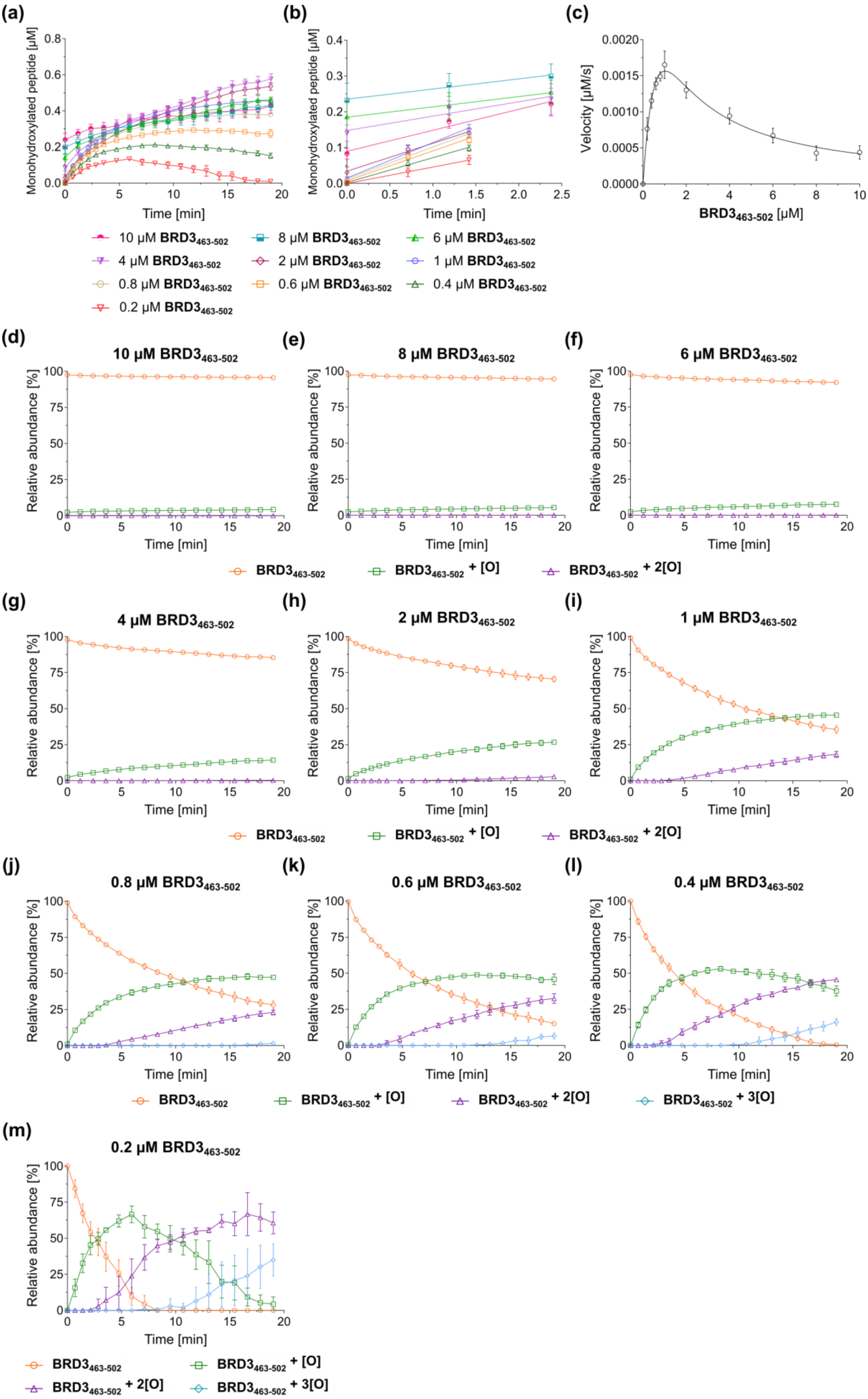
(a)
(b)
(c)
Monohydroxylated peptide [µM]
Time [min]
Velocity [µM/s]
BRD3$_{463-502}$ [µM]
10 µM BRD3$_{463-502}$
8 µM BRD3$_{463-502}$
6 µM BRD3$_{463-502}$
4 µM BRD3$_{463-502}$
2 µM BRD3$_{463-502}$
1 µM BRD3$_{463-502}$
0.8 µM BRD3$_{463-502}$
0.6 µM BRD3$_{463-502}$
0.4 µM BRD3$_{463-502}$
0.2 µM BRD3$_{463-502}$
(d)
10 µM BRD3$_{463-502}$
(e)
8 µM BRD3$_{463-502}$
(f)
6 µM BRD3$_{463-502}$
Relative abundance [%]
Time [min]
BRD3$_{463-502}$
BRD3$_{463-502}$ + [O]
BRD3$_{463-502}$ + 2[O]
(g)
4 µM BRD3$_{463-502}$
(h)
2 µM BRD3$_{463-502}$
(i)
1 µM BRD3$_{463-502}$
(j)
0.8 µM BRD3$_{463-502}$
(k)
0.6 µM BRD3$_{463-502}$
(l)
0.4 µM BRD3$_{463-502}$
BRD3$_{463-502}$ + 3[O]
(m)
0.2 µM BRD3$_{463-502}$

Supplementary Figure S9. Determination of the JMJD6 kinetic parameters for O_2. SPE-MS was used to determine the extent of JMJD6-catalysed mono-hydroxylation of the $BRD4_{511\text{-}550}$ fragment peptide for the specified O_2 concentrations. The concentration of O_2 [%] in the reaction buffer was converted into O_2 [μM] by standard calibration ($y_{[\mu M]} = 9.026 \cdot x_{[\%]}$).[2] Hydroxylation reactions used to determine the JMJD6 kinetic parameters for O_2 were performed at 37 °C and quenched after 60 or 90 s, as indicated. Data are the mean of three independent runs (n = 3; mean ± SD). Note, no evidence for di- (or higher order) hydroxylation of $BRD4_{511\text{-}550}$ was observed under the reaction conditions used. Conditions: His_6-JMJD6 (50 nM), 2OG (200 μM), FAS (2 μM), $BRD4_{511\text{-}550}$ (2 μM), LAA (100 μM) and O_2 (as specified) in Tris buffer (50 mM, pH 7.5) at 37 °C.

(**a**) Initial reaction velocities used for the kinetic analysis of the JMJD6-catalysed mono-hydroxylation of $BRD4_{511\text{-}550}$ under different partial pressures of O_2; (**b**) Michaelis-Menten curve used to determine the kinetic parameters of JMJD6 for O_2. The JMJD6 v_{max}^{app} and K_m^{app} values of JMJD6 for O_2 are 6.5 ± $0.4 \cdot 10^{-3}$ $\mu M \cdot s^{-1}$ and 74.3 ± 13.4 μM, respectively, as determined by non-linear regression.

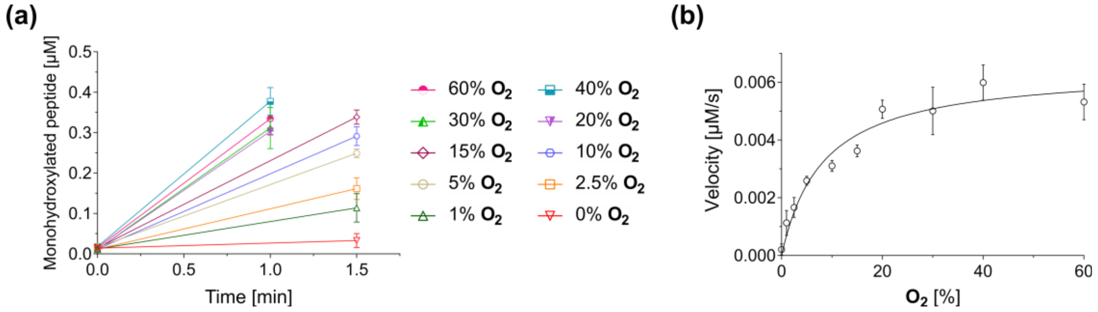

Supplementary Figure S10. Consensus mechanism for 2OG oxygenase catalysis. (**a**) 2OG oxygenases catalyse substrate oxidation (here: hydroxylation) in a manner coupled to the oxidative decarboxylation of 2OG to give succinate and carbon dioxide.[3-5] Note that some 2OG oxygenases catalyse the substrate uncoupled turnover of 2OG to succinate and CO_2, at least in one case leading to a stable ternary enzyme:Fe(III):2OG complex.[6] (**b**) The proposed consensus mechanism for hydroxylation reactions catalysed by 2OG oxygenases.[3] Note, variations on this general catalytic cycle can occur, including with respect to the nature of the 2OG binding mode and the presence of Fe-binding waters, at different stages of catalysis.[7, 8] Mechanistic studies using isotopically labelled O_2 have revealed high levels of O from O_2 is incorporated into the alcohol products of reactions catalysed by human 2OG oxygenases.[9-11] By contrast, (partial) incorporation of isotopically labelled O from water into alcohol products has been observed for reactions catalysed by some bacterial and fungal dioxygenases.[12-17] Note, a consistently high level of a single isotopically labelled O from O_2 into the succinate coproduct has been reported.[18]

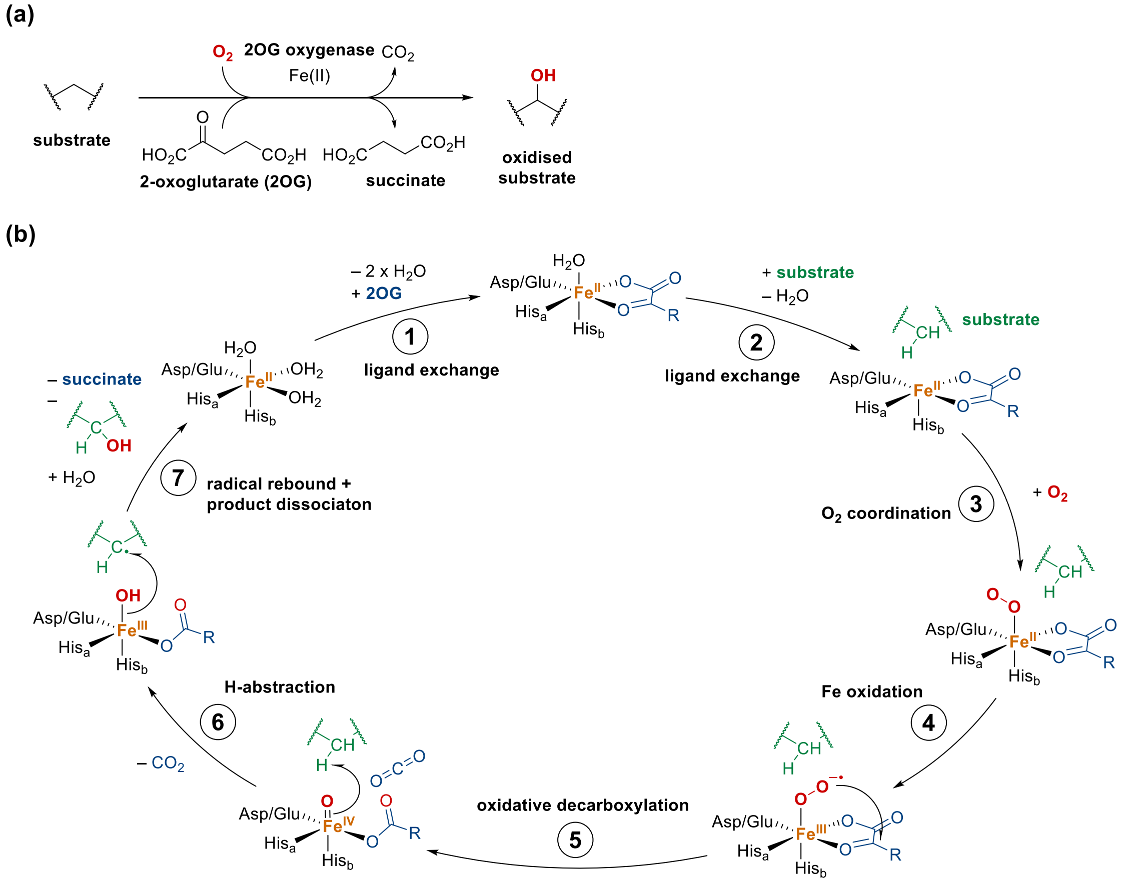

3. References

1. M. Mantri, C. J. Webby, N. D. Loik, R. B. Hamed, M. L. Nielsen, M. A. McDonough, J. S. O. McCullagh, A. Böttger, C. J. Schofield and A. Wolf, *Med. Chem. Commun.*, 2012, **3**, 80-85.

2. R. L. Hancock, N. Masson, K. Dunne, E. Flashman and A. Kawamura, *ACS Chem. Biol.*, 2017, **12**, 1011-1019.

3. S. Martinez and R. P. Hausinger, *J. Biol. Chem.*, 2015, **290**, 20702-20711.

4. K. S. Hewitson, N. Granatino, R. W. D. Welford, M. A. McDonough and C. J. Schofield, *Philos. Trans. R. Soc. A*, 2005, **363**, 807-828.

5. C. J. Schofield and R. Hausinger, *2-Oxoglutarate-Dependent Oxygenases*, The Royal Society of Chemistry, 2015.

6. G. Fiorini, S. A. Marshall, W. D. Figg, W. K. Myers, L. Brewitz and C. J. Schofield, *Sci. Rep.*, 2024, **14**, 26162.

7. M. A. McDonough, C. Loenarz, R. Chowdhury, I. J. Clifton and C. J. Schofield, *Curr. Opin. Struct. Biol.*, 2010, **20**, 659-672.

8. A. Brasnett, I. Pfeffer, L. Brewitz, R. Chowdhury, Y. Nakashima, A. Tumber, M. A. McDonough and C. J. Schofield, *Angew. Chem. Int. Ed.*, 2021, **60**, 14657-14663.

9. E. Holme, G. Lindstedt, S. Lindstedt and M. Tofft, *J. Biol. Chem.*, 1971, **246**, 3314-3319.

10. W. Min, T. P. Begley, J. Myllyharju and K. I. Kivirikko, *Bioorg. Chem.*, 2000, **28**, 261-265.

11. L. A. McNeill, K. S. Hewitson, J. M. Gleadle, L. E. Horsfall, N. J. Oldham, P. H. Maxwell, C. W. Pugh, P. J. Ratcliffe and C. J. Schofield, *Bioorg. Med. Chem. Lett.*, 2002, **12**, 1547-1550.

12. J. E. Baldwin, R. M. Adlington, N. P. Crouch and I. A. C. Pereira, *Tetrahedron*, 1993, **49**, 7499-7518.

13. J. E. Baldwin, R. M. Adlington, N. P. Crouch, I. A. C. Pereira, R. T. Aplin and C. Robinson, *J. Chem. Soc., Chem. Commun.*, 1993, 105-108.

14. B. Lindblad, G. Lindstedt and S. Lindstedt, *J. Am. Chem. Soc.*, 1970, **92**, 7446-7449.

15. P. J. Sabourin and L. L. Bieber, *J. Biol. Chem.*, 1982, **257**, 7468-7471.

16. Y. Kikuchi, Y. Suzuki and N. Tamiya, *Biochem. J.*, 1983, **213**, 507-512.

17. J. E. Baldwin, R. M. Adlington, N. P. Crouch and C. J. Schofield, *Tetrahedron*, 1988, **44**, 643-650.

18. R. W. D. Welford, J. M. Kirkpatrick, L. A. McNeill, M. Puri, N. J. Oldham and C. J. Schofield, *FEBS Lett.*, 2005, **579**, 5170-5174.
